# The earliest elephant-bone tool from Europe: An unexpected raw material for precision knapping of Acheulean handaxes

**DOI:** 10.1126/sciadv.ady1390

**Published:** 2026-01-21

**Authors:** Simon A. Parfitt, Silvia M. Bello

**Affiliations:** ^1^ Institute of Archaeology, University College London, 31-34 Gordon Square, London WC1H 0PY, UK.; ^2^ Centre for Human Evolution Research, Natural History Museum, Cromwell Road, London SW7 5BD, UK.

## Abstract

Organic knapping tools made from bone, antler, and wood were essential to early human toolkits but are rarely preserved in the archeological record. The earliest known soft hammers, dating to ~480,000 years ago, come from Boxgrove (UK), where modified antlers and large mammal bones were used alongside flint hard hammers. These tools facilitated complex knapping techniques, such as platform preparation and tranchet flake removal, contributing to the production of finely worked ovate handaxes typical of the Boxgrove Acheulean industry. This study presents a cortical bone fragment from an elephant, deliberately shaped into a percussor for resharpening flint tools. It represents the earliest known use of elephant bone in Europe and the first documented case of its use as a knapping hammer. Reconstructing its life history offers further insights into Middle Pleistocene hominin technological adaptations, resourcefulness, and survival strategies that enabled humans to endure harsh northern environments.

## Teaser

Europe’s earliest elephant-bone tool sheds light on 500,000-year-old human innovation and cognitive abilities.

## INTRODUCTION

During the early Middle Pleistocene in Europe, hominins encountered two of the largest proboscidean species known: the steppe mammoth *Mammuthus trogontherii* and the straight-tusked elephant *Palaeoloxodon antiquus*. These species, estimated to weigh around 10 tonnes each (*1*, *2*), presented formidable challenges and opportunities for early humans. The hunting of proboscideans by early humans may also have played a critical role in several extinctions over extended periods during the Quaternary and across different continents (*3*, *4*). Whether hunted or scavenged (*5*–*7*), early humans had access to the prodigious resources of a proboscidean carcass, including skin, foot pads, chyme (gut contents), fat, meat, and other edible tissues (*8*–*11*). Elephant bones, teeth, and tusks also served as valuable raw materials. A single 10-tonne straight-tusked elephant, for instance, could yield up to 4200 kg of edible matter (*6*), with bones weighing as much as 2000 kg and individual tusks reaching 150 kg.

Archeological evidence indicates that early humans extensively used elephant bones and tusks throughout the Paleolithic period for a wide range of purposes. Elephant bones were a crucial nutritional resource, rich in fat stored as bone marrow within medullary cavities and grease in the spongy bone (*12*). Moreover, elephant osseous materials served as essential resources for toolmaking, shelter construction, artistic expression, and ornaments, as well as a source of fuel (*13*). Complex and composite tools, along with artwork, musical instruments, and ornaments made from mammoth bones and tusks, are hallmarks of Upper Paleolithic cultures in Europe (*13*). Upper Paleolithic industries associated with some of the first anatomically modern humans in Europe include objects made of mammoth ivory, such as the earliest known wind instruments (*14*), a boomerang (*15*), and portable art (*16*, *17*) including an articulated puppet or “(i)doll” (*18*). Artifacts made of elephant bone and ivory were widespread across Europe until the demise of the mammoth at the end of the Last Cold Stage (*3*).

Mammoth bones were also used in complex structures, whether for utilitarian or ritual purposes (*19*), particularly in the periglacial regions of eastern Europe during the Last Cold Stage (*20*, *21*). The earliest known mammoth bone structures are the early Middle Paleolithic (~190 kya) “bone stacks” associated with early Neanderthal occupation levels within the La Cotte de St Brelade ravine, located in Jersey, an island in the English Channel (*22*–*27*). The burning of elephant bone at the site, along with the relatively treeless landscape at the time of the occupations, suggests that the stacks of mammoth bones may have served as stockpiles of fuel for later use (*28*, *29*).

The use of elephant bone as tools has an early Lower Paleolithic origin in East Africa, at Olduvai (Tanzania), where flaked and edge-polished proboscidean cortical bones have been extensively studied and reinterpreted over the years (*30*–*35*). Recent excavations at Olduvai have uncovered an assemblage of elephant and hippopotamus limb bone fragments shaped by knapping from a single artifact-bearing horizon dating to 1.5 Ma (*36*). The assemblage includes massive, elongated implements made from thick proboscidean cortical bone fragments, up to 38 cm in length, with unifacial and bifacial chipping along the margins. This flaking produced concave edges that may have functioned as scraping implements, although de la Torre *et al.* (*36*) propose that they were “used in percussive and compressive activities, potentially related to the butchery of hippopotamus carcasses that attracted hominins to the area.”

**Fig. 1.**
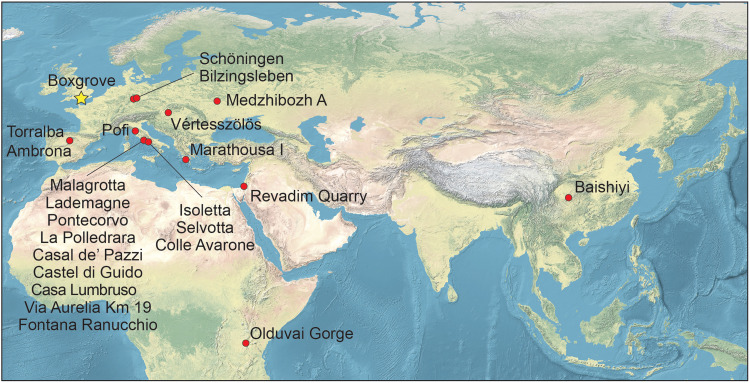
Map of Lower Paleolithic sites with published elephant-bone tools. While bone tools from several sites have been studied using technological, taphonomic, and use-wear analyses, others remain more enigmatic—such as the minute, chipped ivory objects from Medzhibozh A (Ukraine) and the polished ivory from Schöningen (Germany), as well as more controversial cases where identification is based solely on morphology—often at sites lacking associated lithic artifacts, such as Baishiyi (China). At other sites, including Torralba and Ambrona, the interpretation of modified elephant bones as tools has also been questioned (see table S1 for details).

Beyond Olduvai, Lower Paleolithic elephant-bone tools are documented from fewer than 22 sites, mostly located in Europe (Fig. 1 and table S1). These tools vary widely in sophistication, from rudimentary cutting and scraping implements made from unmodified cortical pieces identified as tools from use-wear (*37*) to intentionally flaked cortical bone (*36*) and finely worked Acheulean bone handaxes (*38*, *39*), the latter clustering at sites near Rome, Italy (*40*, *41*).

The use of elephant bones in the manufacture of Middle Paleolithic (Mousterian) lithic tools was originally proposed for pitted and grooved mammoth foot bones found at Kosh-Koba, Crimea (*42*). Semenov (*42*) interpreted these flat supports as being used in two ways: for percussion retouching of lithic scrapers and points and as anvils to support cores during flaking. The interpretation of the Kosh-Koba–modified mammoth foot bones as anvils, however, has been questioned by Haynes (*43*), who suggested that the damage more closely resembles carnivore chewing. Although Semenov also mentions numerous retouchers made from equid diaphyses at the site, Haynes’s suggestion is compelling, especially considering the presence of spotted hyaenas in the Kosh-Koba faunal assemblage.

Elephant bones interpreted as knapping implements used in stone tool production have been identified at only two Lower Paleolithic sites: Olduvai in Tanzania and Marathousa in Greece. However, the evidence supporting their use in this capacity remains ambiguous. At Olduvai Gorge, notable examples include an elephant patella (FLKII no. 884) and a giraffid astragalus (BKII no. 2933) from Bed II, dated to approximately 1.7 to 1.15 Ma. These specimens have been variously interpreted as anvils (*44*, *45*) or hammers (*32*–*34*) used for working lithic tools; striking wedges to split bones, fruits, or wood (*32*); and even as supports for securing skins during piercing with stone awls in hide working activities (*30*). An alternative explanation was proposed by Pante *et al.* (*35*), who highlighted the similarity between the punctures on the elephant patella and crocodile feeding marks, as previously documented by Njau and Blumenschine (*46*), Njau and Gilbert (*47*), and Sahle *et al.* (*48*). While Pante *et al.* rejected the identification of the Bed II elephant patella as a tool, they endorsed Leakey and Roe’s (*45*) earlier suggestion that the pits observed on the articular surface of another elephant astragalus (no. 3109) from site JK, Bed III (1.15 to 0.93 Ma), were likely the result of their use as an anvil for bipolar percussion. Nevertheless, this interpretation remains equivocal, as the same surface also bears marks consistent with carnivore tooth impressions.

The second example of a potential knapping percussor made from elephant bone comes from the Lower Paleolithic site of Marathousa I (~478,000 to 424,000 years ago) in the Megalopolis Basin, Greece. This specimen is identified as a fragment of elephant limb-bone shaft, bearing cut marks, flake scars, and percussion damage at one end (*49*). However, the authors note the limited presence of soft hammer flakes within the Marathousa lithic assemblage, an expected by-product if bone had been routinely used in stone tool production. Hence, the functional interpretation of this fragment remains inconclusive, and further analysis, including high-resolution imaging of the surface modifications, is required to clarify its use (*49*).

The examples from Olduvai and Marathousa underscore the difficulties in distinguishing early instances of bone tool use from taphonomic modifications caused by nonhominin agents. They highlight the interpretive challenges posed by the often-subtle overlap between natural damage (such as carnivore chewing) and marks potentially resulting from the use of bones as knapping tools (*50*).

Here, we report the discovery of a knapping tool made on an elephant bone from a ~480,000-year-old Acheulean context at Boxgrove, UK. Through an analysis of its archeological and landscape context, production process, and utilization patterns, we show how the bone was intentionally flaked before being transported to a new location, where it was used as a soft hammer to resharpen lithic tools. This find marks the earliest known instance of elephant bone being used as a raw material in Europe, as well as the earliest unambiguous reported use of elephant bone as a knapping percussor. We explore the implications of our findings for gaining additional insights into the technological, cognitive, and behavioral adaptations of hominins at Boxgrove, set within the broader environmental and cultural context of the early Middle Pleistocene.

## RESULTS

### Archeological context

**Fig. 2.**
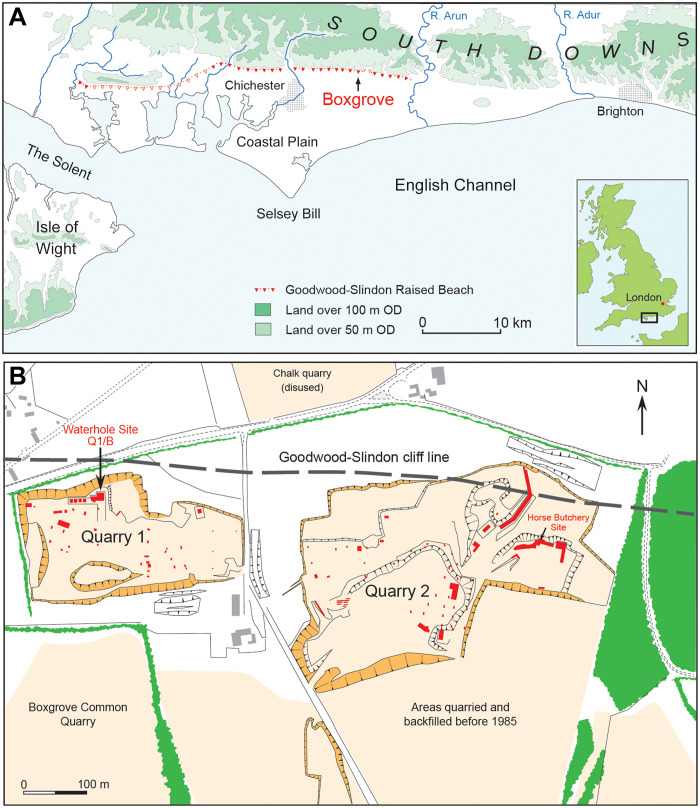
Location maps. (**A**) Location of Boxgrove in relation to the Goodwood-Slindon (40 m) Raised Beach. (**B**) Extent of quarrying at Boxgrove and the principal archeological sites (red).

Boxgrove is one of the most comprehensively excavated Lower Paleolithic open air sites, with archeology and environmental evidence providing unique insights into early Middle Pleistocene hominin behavior during a period of major climate change in northwestern Europe. The archeological deposits at Boxgrove were uncovered in sand and gravel quarries, situated along an ancient shoreline with cliffs to the north of a large marine embayment, spanning what is now the southern part of the English Channel (Fig. 2A). This site has yielded a wealth of stone artifacts, together with hominin remains and other well-preserved fossils, including those of mammals ranging in size from pipistrelle bats to elephants, as well as birds, reptiles, amphibians, fishes, mollusks, foraminifera, and ostracods (*51*–*55*). These occur within a 25-m-thick geological sequence spanning the transition from an interglacial nearshore marine environment to a resource-rich plain with spring-fed ponds into a major cold phase during which gravel and clay were transported downslope from higher ground above the site by periglacial processes. Dating of the succession is based primarily on mammalian biostratigraphy (*56*), which correlates the marine deposits to the terminal part of Marine Isotope Stage (MIS) 13 (533 to 478 ka) and the overlying terrestrial sediments with the transition from interglacial conditions to a major cold stage of MIS 12 (478 to 424 ka).

The archeological investigations identified a succession of horizons that reflect recurrent episodes of hominin activity in a landscape that developed from a beach and intertidal mudflat to a stable landsurface (paleosol) with springs and ponds, followed by a period of cooling climate with the gradual influx of colluvium from the cliff. As well as rare hominin remains [including a tibia and two incisors: (*57*–*59*)], the archeological evidence comprises a rich lithic industry, large mammal remains with cut marks and evidence of marrow and grease processing, and an exceptional assemblage of antler and bone knapping tools (*60*–*63*).

**Fig. 3.**
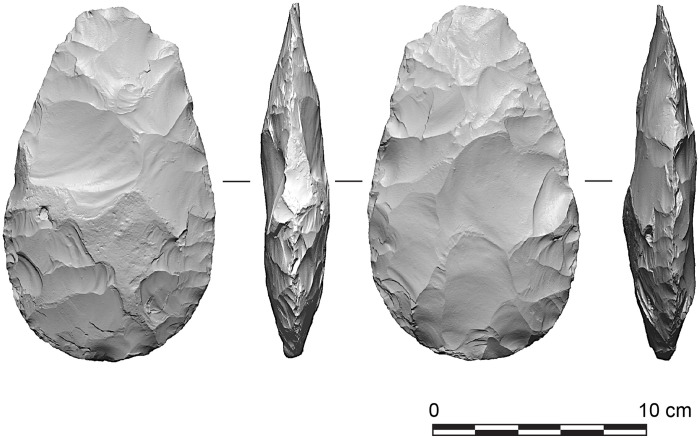
Handaxe from the Boxgrove paleosol horizon (locality Q2/GTP 17).

One of the most notable excavated areas is the Q1/B “Waterhole Site,” situated near the cliff line in the northwestern part of Quarry 1 (Fig. 2B). At this locality, a spring and freshwater ponds provided a unique environment that attracted animals and intermitted visits by hominins, who discarded considerable quantities of finished and unfinished handaxes and butchered animal remains at the site (*64*). In all, more than 459 finished handaxes and rough-outs were excavated at the waterhole site alone (*65*, *66*), and these include numerous precisely shaped examples (Fig. 3).

Evidence for the handaxe manufacturing processes stems from exceptionally well-preserved knapping scatters found at this site and other locations and horizons within the quarry complex (*53*). These scatters represent short episodes of tool manufacture and resharpening in minimally disturbed contexts. Landscape-scale excavations have revealed a multistage process of raw material acquisition and tool production. At the foot of the cliff, hammerstones were collected, and flint nodules were tested and reduced to early-stage handaxe roughouts. Further from the cliff, more complete sequences of tool manufacture have been documented, including nodule reduction, handaxe and flake tool manufacture, and resharpening of dulled edges at butchery sites (*54*). Discrete knapping scatters associated with both the shaping of handaxes and the resharpening of flint cutting tools have also been documented (*53*).

A detailed use-wear analysis of the Boxgrove handaxes conducted by Mitchell (*67*) demonstrated that the examined sample was used predominantly—if not exclusively—for butchery. Although it remains theoretically possible that handaxes served a broader range of functions, the use-wear evidence from Boxgrove, including both micro- and macrotraces, does not support activities such as woodworking or digging. These heavier-duty tasks typically leave distinctive wear patterns, which are notably absent from the Boxgrove handaxes.

The Boxgrove handaxes are notable for their high degree of technical refinement and standardization (*68*–*70*). They exhibit a preferred ovate shape with straight cutting edges that were often skillfully finished by the removal of tranchet flakes along the handaxe tip, resulting in an exceptionally sharp and straight cutting edge (*69*). Because of the well-preserved discrete knapping scatters, each representing different stages of the manufacturing process, it is possible to reconstruct the knapping strategies used in producing the Boxgrove handaxes with a high level of detail (*53*, *54*). The refinement of the Boxgrove handaxes was achieved by the switch from hard (flint) hammers to soft (antler and bone) hammers at an early stage in the manufacturing process and particularly during the intense final shaping and thinning stages (*71*). Flakes from this part of the knapping sequence provide the first direct archeological evidence for platform preparation in bifacial thinning and the production of technically demanding tranchet tips (*69*, *70*, *72*). The use of both hard and soft hammers required distinct dexterous motor skills, along with advanced visual-motor coordination and forward planning, to shape handaxes from amorphous nodules into the desired forms.

An exceptional aspect of the Boxgrove artifact assemblage is the range of osseous knapping hammers that include varied types of bone percussors and at least three antler hammers (*60**,* *72**,* *73*). These tools played a crucial role in the knapping process that allowed the Boxgrove hominins to shape, maintain, and enhance the effectiveness of the flint handaxes.

### Elephant remains from Boxgrove

Elephant bones are exceptionally rare at Boxgrove. Despite extensive excavations and surface prospecting across faunally rich horizons—spanning up to 900 m laterally along the cliff’s base and extending 200 m from the cliff line—only 24 heavily fragmented elephant bones, representing a minimum of six elements, have been found at the site (table S2).

**Fig. 4.**
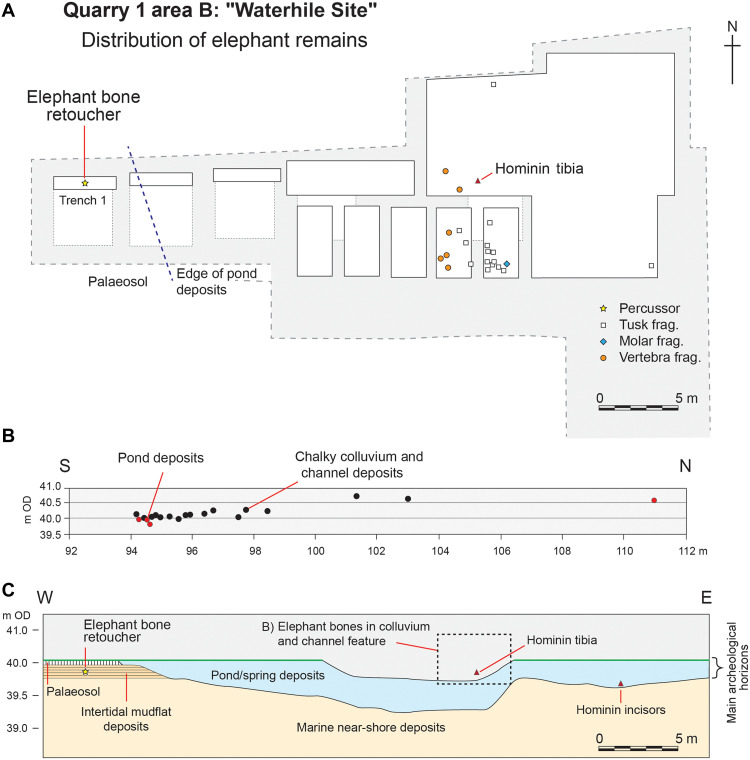
The spatial distribution of elephant remains at the Waterhole Site (Q1/B). (**A**) Plan showing the distribution of element types. frag., fragments. (**B**) Vertical distribution of elephant remains plotted on a north-south profile. (**C**) Simplified geological section through the excavated deposits at the Waterhole Site. The Pleistocene deposits rest on chalk bedrock (not shown) at an elevation of ~35 m Ordnance Datum (OD), which is broadly equivalent to mean sea level, and the freshwater deposits are overlain by ~10 m of soliflucted gravels deposited under periglacial conditions. Note: The elephant-bone tool derives from intertidal deposits that are stratigraphically lower than the colluvium and channel feature containing other elephant bones. This indicates that the tool represents a separate depositional event and a distinct occurrence from the later material.

Most of the specimens are from the Waterhole Site (Q1/B) in Quarry 1 (Fig. 4). Stratigraphically, these specimens are associated with the fill of a shallow channel and the basal horizons of the colluvial sequence that overlies the main body of freshwater pond and spring deposits (Fig. 4C). The deposits containing the elephant bones also yielded a hominin tibia together with occasional flint artifacts (*57*). The elephant remains in this group consist of a piece of an enamel plate from a cheek tooth, along with 14 weathered tusk fragments (none larger than 43 mm) and six fragments of an unfused vertebral epiphysis (the largest of which is 119 mm in length).

The vertical distribution of the elephant remains (Fig. 4B) aligns with the dip of the colluvial deposits, which formed mounds and fans (Unit 8a) as the cliff eroded over time. Although no refitting pieces could be identified between any of the fragments, they most likely originated from a small segment of a tusk, weighing no more than 30 g, which had weathered and disintegrated on the landsurface, and a single vertebral epiphysis that underwent a similar process before being dispersed down the slope and into the channel feature (Unit 8ac). Three fragments from the upper part of the underlying freshwater deposit (Unit 4) were probably incorporated into the lower deposits due to mixing caused by either soft sediment deformation or bioturbation. An isolated tusk fragment (NHMUK PV M 119823) exhibits a similar condition to the other pieces, suggesting that it may belong to the same group of shattered tusk fragments found in the basal colluvial sediments. The fractured tip of a tusk (NHMUK PV M 119836) from a lower level in the northern part of the site may originate from a different tusk. In addition, sieving of the paleosol horizon (Unit 4c) to the west of the channel feature yielded a small piece of elephant tooth enamel.

The elephant bone percussor (NHMUK PV UNREG 4339; field number Q1/B F A317) was excavated from a lower level in the westernmost trench (Trench 1) in Q1/B, within a 0.5-m-thick sequence of intertidal laminated silts and sands (Slindon Silt Formation). This horizon contains micropaleontological evidence (ostracods, abundant earthworm granules, and slug plates) for reduced salinity and the proximity of drier ground and a freshwater input to the saltwater lagoon. Excavations of the intertidal deposits in Trench 1 covered an area of 5.5 m^2^ and yielded few additional finds. Among them were fragments of a cervid sacrum and the neural arch from a large mammal vertebra, neither of which exhibited discernible traces of hominin alterations.

The only elephant bone not originating from Q1/B is a shattered fragment of cortical bone (weighing just more than 1 kg). It was found in spoil resulting from quarrying operations, where the dragline had penetrated the surface of marine deposits ~180 m southeast of Q1/B, Trench 1. Despite careful searches of the surrounding area and the excavation of a trench (GTP 33) in the vicinity, no additional pieces were found.

### The elephant-bone knapping tool

**Fig. 5.**
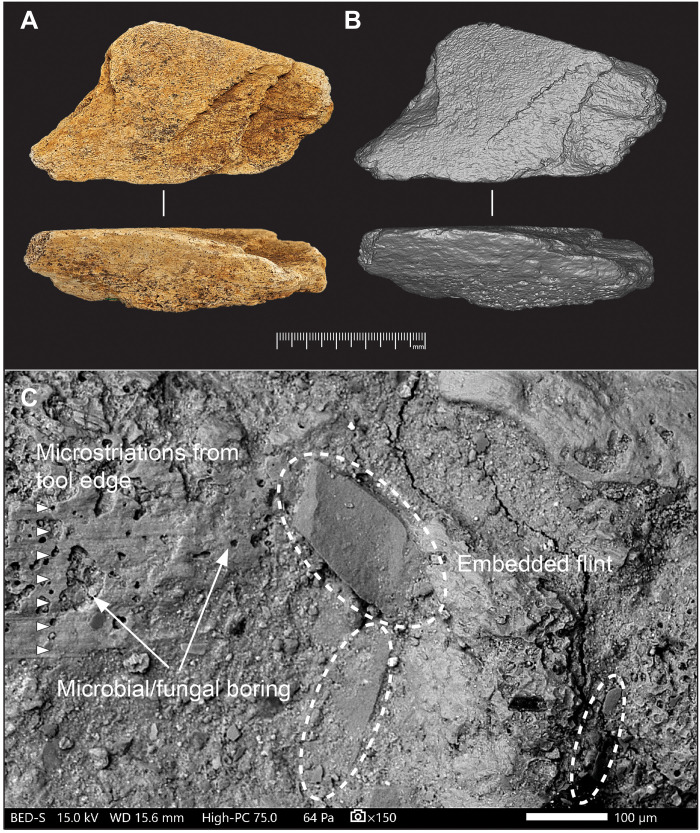
Elephant-bone tool (NHMUK PV UNREG 4339) from intertidal deposits at the Boxgrove Waterhole Site (Q1/B). (**A**) Photographs and (**B**) three-dimensional (3D) surface model images. The outer cortical surface shows indentations resulting from its use as a flint-knapping tool. Side views illustrate the invasive flaking of the cortical surface. See figs. S1 and S2 for other views. (**C**) Scanning electron microscope (SEM) micrographs of features inflicted during use as a knapping tool, including microstriations and embedded flint chips (examples highlighted by dashed ovals), alongside evidence of postdepositional bioerosion. Patches of fine sand and silt adhere to the surface, filling, or partially obscuring depressions. Copyright of the Trustees of the Natural History Museum, London.

NHMUK PV UNREG 4339 is a tabular fragment of elephant cortical bone, excavated from the intertidal deposits at Quarry 1/B. It is roughly triangular in shape, measuring 109 mm in length, 58 mm in maximum width, and 30 mm in thickness (Fig. 5, A and B, and fig. S1), with a cross section closely resembling that of a right trapezoid. The outer compact bone is ~15 mm thick, grading into trabecular bone toward the inner surface. Overall, the edges and surfaces are unabraded and well preserved, with only a few small areas with accidental damage that occurred during excavation.

Examination of the surface of the bone under the scanning electron microscope (SEM) reveals circular holes and channels ~10 μm in diameter (Fig. 5C). Associated with this superficial modification, further signs of postburial degradation are evident where excavation damage has removed the surface layer, exposing the bone’s interior, which displays a soft, “chalky” texture overlying a denser core. Previous histological analysis of bones from the intertidal deposits at Boxgrove had shown that, while the internal “core” retains typical bone histology, the outer layer exhibits extensive and pervasive tunneling, which destroyed much of the original histological structure (*74*). This type of degradation is often attributed to fungal activity, although bacterial activity may also have contributed to the bone’s structural deterioration (*75**–**80*). Despite the loss of the original histological structure in the outer cortex layer, the surfaces of bones from the Boxgrove intertidal deposits are generally well preserved. The two-layer preservation pattern and surface boring observed in the elephant bone from Q1/B, along with the characteristic light-brown coloration from iron oxides and black patches from manganese dioxide, are consistent with the preservation patterns of bones found in other intertidal sediments at Boxgrove. At a microscopic level, many of the smaller-scale features are fully or partially filled with sediment, which was intentionally left in place to preserve delicate microscopic details.

Specimen NHMUK PV UNREG 4339 displays typical characteristics of elephant bones: a thick layer of predominantly compact cortex, which grades into longitudinally aligned spongy bone toward its inner surface; the cortical surface preserves the original reticular “grain” structure, which, in combination with the overall thickness of the piece, is also typical of elephant bones. Despite the cortical thickness suggesting that the tool was fashioned from a large, thick-walled elephant bone, it remains uncertain whether the piece originated from a limb bone or another part of the elephant’s skeleton. Similarly, the elephant remains from Boxgrove are currently inadequate to identify the specific proboscidean species that inhabited the area. Nonetheless, on the basis of findings from other early Middle Pleistocene sites in Europe (*81*–*83*), the two probable candidates are the steppe mammoth *M. trogontherii* and the straight-tusked elephant *P. antiquus*.

**Fig. 6.**
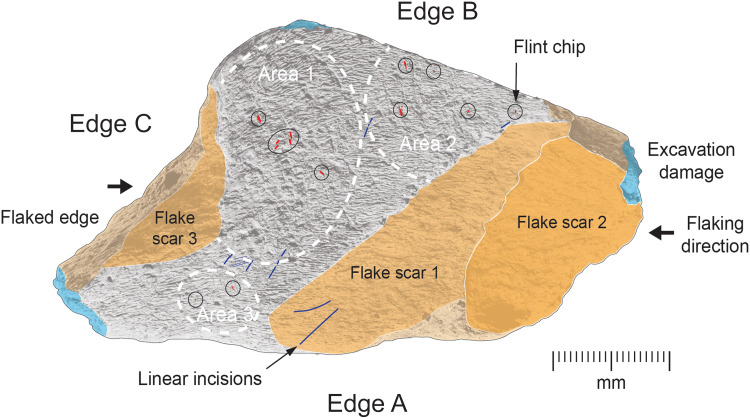
Postmortem modifications. Cortical surface showing breakage planes (Edges A and C), flake scars 1 to 3, anthropic linear incisions (in blue), and embedded flint fragments (in red, circled in black). Knapping marks are concentrated in three areas (areas 1 to 3). See figs. S3 to S5 for additional images of the surface features. Copyright of the Trustees of the Natural History Museum, London.

Two stages of fracture can be identified. The first phase involved breaking the bone to produce a “blank” likely similar in size to the finished tool (Fig. 6 and fig. S2). The longest margin (A) displays a sharp, linear edge with a planar fracture surface that is slightly curved along its length and angled inward at ~45° from the cortical surface. The opposing margin (B) is also planar and gently curved, oriented at a right angle to the cortical face. The break surfaces along margins A and B are truncated by flake scars from the second stage of breakage. The shorter margin (C) appears to have been further modified during the second stage of breakage, resulting in a rougher, irregular fracture surface incorporating a flake removal from the cortical surface. Although it is not possible to determine whether the blank was intentionally fractured, the curving profiles of the margins suggest that the breakage occurred while the bone was still “green” (i.e., unweathered).

The second phase of breakage involved the removal of flakes from opposing ends of the piece through direct percussion, detaching them from the outer (cortical) face (fig. S2). These removals are evidenced by remnant flake scars from at least three blows. At one end, the first flake removal (flake scar 1) consists of a relatively shallow scar with an irregular hinge termination, grading into a feather edge along its length. The distal end of the detached flake would have been about 50 mm in width, although its length is unknown, as the proximal end was removed by a subsequent flake removal. The second flake removal from this end (flake scar 2) was struck from the same direction, detaching a thicker (~7 mm) invasive flake at least 40 mm wide, with a curved hinge fracture. The flake scar at the opposite end (flake scar 3) is also invasive, terminating in a hinge fracture with a planar profile. The detached flake would have been at least 30 mm wide and 6 mm thick at its distal end. The bone edge bordering this flake removal (Edge C) appears to have been further modified by additional blows, detaching irregular bone fragments.

These flake removals appear to have been intentional and related to shaping rather than to marrow extraction or postdepositional processes. Several observations support this interpretation. First, the large invasive flakes are located at the ends of the fragment and were removed from preexisting fracture edges rather than directly from the cortical exterior, suggesting a deliberate reduction in length or shaping rather than flaking from percussion aimed at accessing marrow, which would typically produce features radiating from the cortical surface toward the medullary cavity. Second, the opposing removal of flake scar 3, oriented parallel to the cortical surface, is unlikely to result from trampling. If trampling had occurred, particularly on a rocky substrate, then we would expect associated scratch marks or characteristic surface damages, which are absent here.

In addition to these observations about flake creation, there is evidence that the flake removals predate the bone’s use as a knapping hammer. This is indicated by knapping marks that extend across the hinge terminations of flake scars 1 and 3.

A further set of features made before the bone’s use as a tool includes five fine, linear incisions running diagonally across the surface between areas 1 and 3 and within area 2, the longest of which has a truncated length of 5 mm. These incisions exhibit V-shaped, symmetrical cross sections with longitudinal internal striations, indicating that they were made with a sharp stone edge (fig. S5, F and I). Their morphology is consistent with typical slicing cut marks (*84**,* *85*). Between two of the parallel incisions, a broad zone of shallower, scrape-like marks is visible. The presence of pitting and scores superimposed on these features indicates that they were made before the bone was used as a knapping tool. Additional shallow scrape marks are also present within the intensely pitted area near the center of the cortical surface. While the interpretation of these incisions and scrapes remains uncertain, they may reflect the removal of soft tissues in preparation of the bone to be used as a tool, a preparatory stage also observed in other bone soft hammers from Boxgrove and Lower Paleolithic retouchers, such as those from Schöningen (*86*).

In addition to these preuse modifications, two longer linear grooves (6 and 8 mm) with similar morphologies are present on the flaked surface of the bone within flake scar 1. These scratches were scored into the surface by a sharp object after flaking had occurred. Despite their resemblance to cut marks, the specific causal process responsible for these marks cannot be determined.

**Fig. 7.**
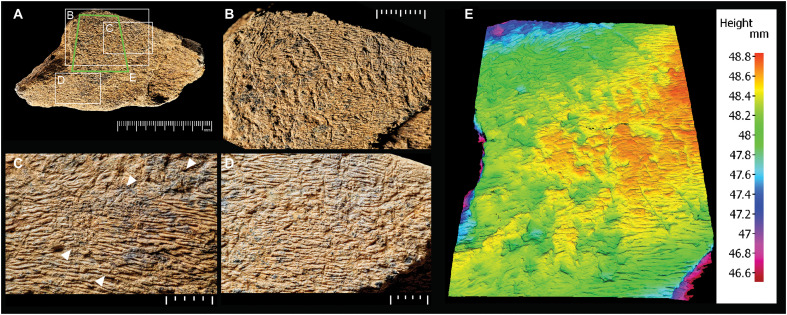
Details of surface modifications. (**A** to **D**) Photographs of battered areas of the elephant cortical bone, showing the distribution of the pits and scores, which are clustered in three areas: (B) area 1 (left) and area 2 (right); (C) area 3; (D) close-up of area 2. The oblique incisions visible in (C) marked by arrows cross areas 1 and 3, where they are truncated by pits and scores. (**E**) 3D surface model showing the unmodified texture of the cortical surface intersected by pits and scores. Scale divisions in millimeters. Copyright of the Trustees of the Natural History Museum, London.

Evidence for how the bone was used as a tool can be inferred from areas of intensely battered bone with numerous superimposed angular pits, scores, and striations (Fig. 7 and figs. S3 to S5). Most of the battering is located within the widest part of the cortical surface, but the features are scattered across an area of about 2000 mm^2^, bounded by the flake scars and the longer marginal break surfaces.

The battering marks are concentrated in three areas (Fig. 6). Area 1 has a high concentration of angular pits interspersed with parallel scores. A smaller overlapping zone (area 2) is characterized by parallel scores of varying depths. The third area (area 3) consists of shallower, less well-defined parallel scores and areas of roughened bone that are barely perceptible under the light microscope (fig. S5C).

**Fig. 8.**
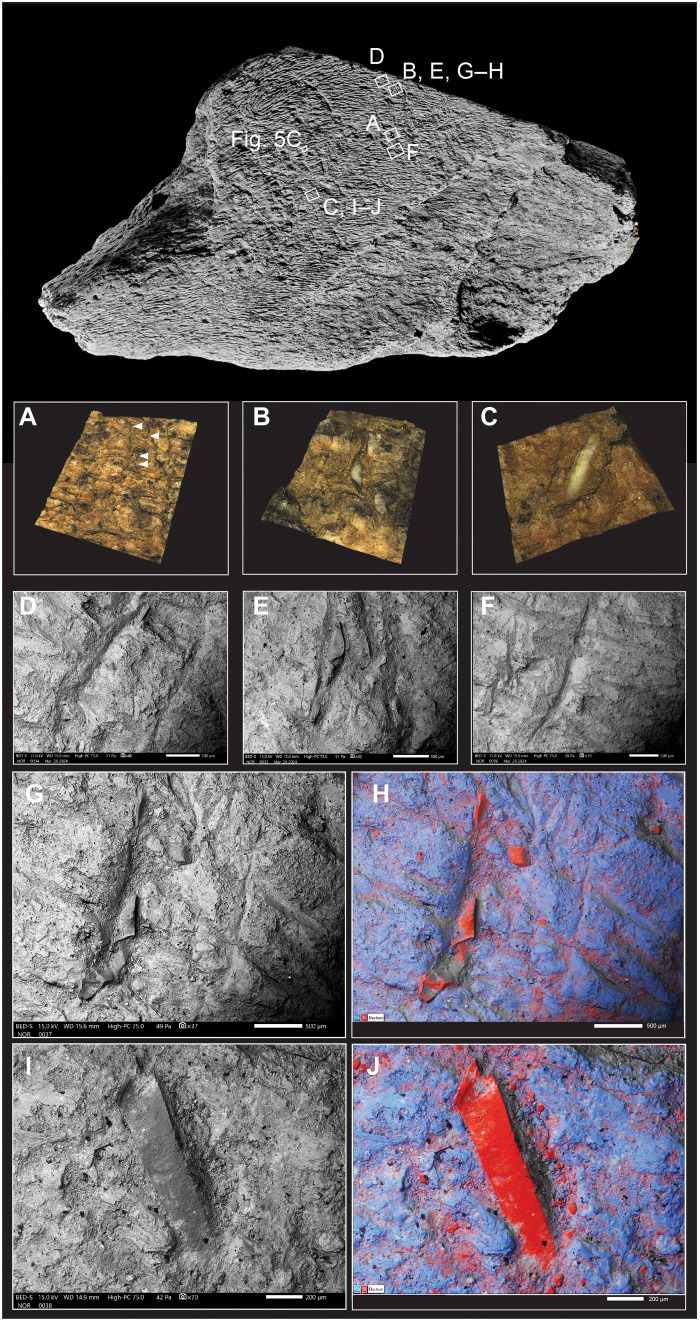
Scores, pits, and embedded flint chips in areas 1 and 2. Features illustrated with 3D surface models (**A** to **C**) and SEM micrographs (**D** to **J**). Arrows indicate the smallest microchips. For embedded flint chips, the smallest examples are indicated by arrows (A). SEM micrographs [(G) and (I)] and EDX/SEM images [(H) and (J)] of flint chips (red: silicon) embedded in bone (blue: calcium). Gray areas in the EDX images are zones hidden (shadowed) from the detector. Copyright of the Trustees of the Natural History Museum, London.

The pits are triangular or roughly circular indentations displaying angular edges and pyramidal cross sections, with diameters of about 1 to 2 mm. The scores are linear indentations with the appearance of chop marks and have a preferred orientation slightly oblique to the long axis of the bone. In plan, they are typically straight or sinuous and up to 8 mm in length. Figure 8 (D and F) illustrates examples of scores in which the internal features are not obscured by sediment. These grooves exhibit asymmetric cross sections resembling a “✓,” with the longer, smoothly angled leading edge contrasted by the opposite side, which displays irregular and roughened surfaces where the impact force has detached part of the bone. In other examples, this trailing edge has a rim of deformed bone pushed forward by the force of the blow. The leading edges of the scores exhibit a consistent polarity on the same side of each feature in the parallel set. However, a few indentations have a transverse orientation, differing from the predominant alignment of most of the scores.

The flatter walls on the leading side of the scores exhibit smooth surfaces, often marked by fine parallel striations that run transverse or slightly oblique to the longer axis of the score. Under the SEM, these microstriations are visible on the bone surface extending beyond the boundaries of the scores on both sides, with those on the leading side merging with the slightly angled striations within the indentation (e.g., Fig. 5C).

Many of the scores contain tiny fragments of embedded lithic debris at the base of the indentations. Fourteen larger pieces were identified under the binocular microscope (Fig. 6), while numerous smaller pieces were observed in the SEM/energy-dispersive x-ray (EDX) images (Fig. 8, H and J). The lithic debris is primarily embedded within the scores and is scattered throughout the three knapping areas (Fig. 6). SEM images reveal that most of the lithic debris is shattered from the impact (e.g., Fig. 8A), and EDX analysis confirms a silicon composition consistent with flint.

## DISCUSSION

### Interpreting how the elephant-bone tool was used

The battering damage containing embedded flint chips on the Boxgrove elephant bone identifies it as a rare category of Lower Paleolithic osseous tool known as a “retoucher” (*87**–**92*). Bone retouchers and percussors were essential knapping tools in the Paleolithic for the production, shaping thinning, and resharpening of stone implements. They have been known since the 1870s (*88*, *89*), but interest in them surged from the 1990s onward, driven by new imaging techniques and their increasing recognition in Paleolithic bone assemblages (*44**,* *50**,* *87**–**92*). Retouchers, typically made from animal bones and teeth, were used to strike or apply precise pressure to the edges of stone tools, such as handaxes and scrapers, to remove small flakes and sharpen or reshape the cutting edge. The use of soft hammers and retouchers allowed for greater control in the knapping process compared to hard hammers, enabling early humans to produce more finely shaped and efficient tools.

Osseous knapping tools can be distinguished from other implements by a specific combination of use traces, such as clusters of punctiform pits, parallel linear scores and gouges, and abrasion caused by contact between the lithic tool edge and the knapping surface (*86*). They may also contain embedded lithic fragments, which can be detected using SEM/EDX images, and analyzed to identify the material being worked. The configuration of impact features on the utilized elephant bone fragment from Boxgrove provides valuable insights into how the tool was used and the specific flint-knapping tasks it performed; such features are shaped by contact with lithic edges, leaving imprints that correspond to the morphology of the lithic tools being worked (*85*).

The imprints on the working area of the Boxgrove elephant bone fragment include pits clustered in area 1, along with linear impressions across all three areas. The linear features vary in shape, with some exhibiting straight edges and others showing more sinuous or stepped platforms. These variations reflect contact with either straighter edges or more irregular, bifacially worked edges, respectively (fig. S6). The pits and scores observed on the Boxgrove elephant bone retoucher likely result from contact with an edge featuring an undulating profile, with pits forming when the percussor makes contact with peaks along the lithic edge. This edge profile is characteristic of bifacially worked lithic tools.

Features associated with the linear impressions reveal both the directionality of the tool’s movement and the angle at which the tool was held relative to the lithic edge being worked. Microscopic examination of the Boxgrove elephant bone percussor reveals the presence of striae associated with knapping marks, which result from the stone tool sliding across the surface of the percussor before becoming embedded in the knapping area (Fig. 5C). These marks, known as tool edge scratches [sensu (*86*, *92*)], commonly appear on the leading edge of the impressions and extend into the pits, scores, and gouges. The tool edge scratches in some cases continue until the percussor abruptly stops, which can deform the trailing edge of the feature. This abrupt stop can create an irregular trailing edge with a torn appearance or push forward a ridge due to the force of the blow (Fig. 8, D and F). Tool edge striae may also extend beyond the impressions if the lithic edge continues to make contact with the freely moving percussor.

The discrete outlines of the knapping features, along with the “plastic” deformation observed along the trailing edge of some linear impressions, suggest that the elephant bone was relatively fresh (“green”), and still retained enough collagen to remain pliable at the time of use. These characteristics contrast with retouchers made with weathered bones, which typically show less regular knapping marks that are often associated with detached “plates” of bone (*86*, *92*). This pattern of damage is consistent with use on green bone, implying that the fragment was utilized relatively soon after the animal’s death—before substantial drying or weathering had occurred.

However, in temperate environments, a lengthy period may elapse between death and the onset of notable weathering or degradation of the bone’s organic components. Thus, while the damage indicates that the bone was worked while still fresh, this does not necessarily imply immediate postmortem use. Rather, it suggests that the bone retained enough elasticity at the time of use to produce the observed patterns. On this basis, we interpret the object as having been used within a relatively limited timeframe. At the Q1/B locality, it was likely used during ongoing knapping activities at a butchery site, after which it was discarded.

**Fig. 9.**
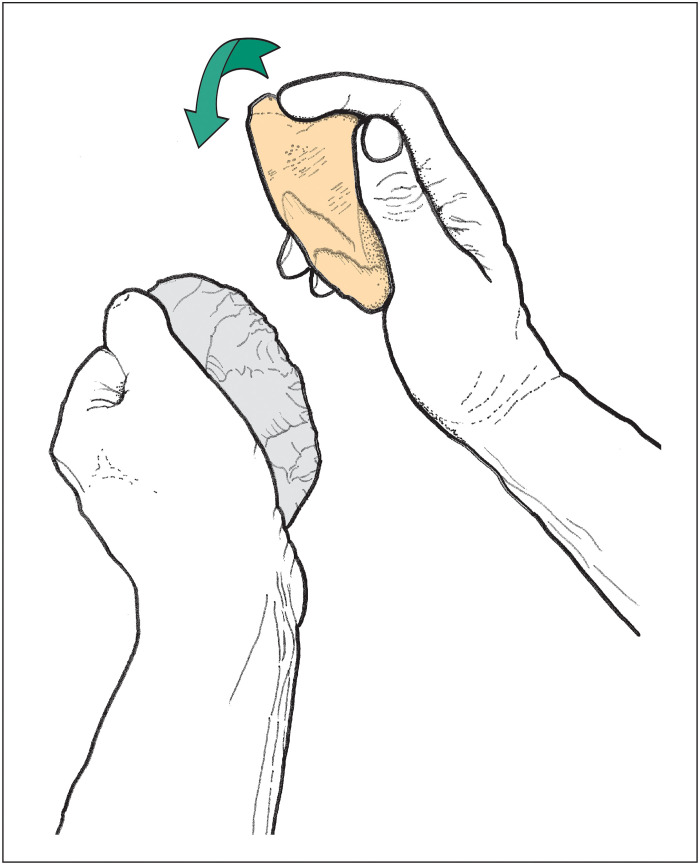
Reconstruction illustrating the use of the elephant-bone knapping hammer to resharpen the edge of a handaxe.

The predominantly parallel to subparallel (oblique) orientation of the knapping scores and the asymmetric profiles of the scores indicate that the bone tool was held in a consistent position and struck repeatedly against the lithic edge with a uniform action and direction to detach flint flakes from the tool edge (Fig. 9). In this configuration, the knapping tool would have been supported by the palm and stabilized by pressure from the encircling fingers and thumb. The occasional longitudinal linear features suggest that some blows were delivered with slight changes in the orientation of the hand.

Another notable feature of the knapping marks on the Boxgrove percussor is the relative shallowness of the impressions, particularly those in area 3. An examination of the damage on other bone knapping hammers from Boxgrove reveals that some, such as the distal humeri of bovids and deer, exhibit extensive battering marks and large flake scars. These features indicate that they were used for forceful blows to detach large flakes, both during the initial shaping of the handaxe blank and in the later thinning stages (*62*). In contrast, the elephant percussor displays relatively shallow knapping marks, suggesting that it was used with lighter blows. This pattern closely resembles that found on lighter bones and diaphysis splinters from Boxgrove, which can only have been used to remove smaller flakes during the final thinning stages of handaxe production or in tasks such as edge modification (*61*). Moreover, the presence of small resharpening flint debris at the find spot of the elephant bone percussor suggests that the percussor was likely used to resharpen the blunted cutting edge of a bifacially worked stone tool, such as a handaxe.

Although it is difficult to determine the exact frequency and duration of its use, the percussor was likely used on multiple occasions for brief resharpening episodes. If each cluster of pits and scores corresponds to a single contact with a lithic edge, then the tool may have been used to deliver more than a hundred blows. This pattern suggests repeated use across multiple sessions, particularly because resharpening a handaxe’s edge typically requires only a few strategic flake removals. The curation and transport of this implement may also explain its isolated recovery, distinct from the sparse and scattered elephant remains found at the site. This possibility raises broader questions about how tools were selected, maintained, and moved across the landscape.

### Manufacture, use, and discard of the elephant-bone knapping tool within the Boxgrove landscape

**Fig. 10.**
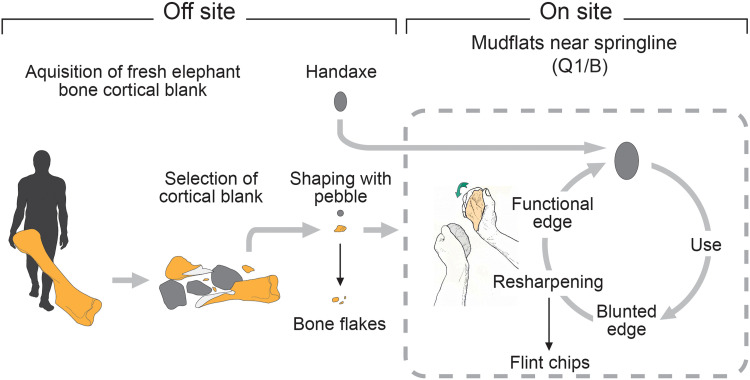
“Life history” of the elephant-bone tool. Schematic diagram illustrating the stages in the manufacture and use of the elephant bone knapping tool, from obtaining the blank and shaping it to create an effective hammer for resharpening lithic tools to its eventual discard or loss. The diagram also highlights the by-products (bone flakes and knapping debris) generated at each stage, which contribute to an archeologically identifiable signature. See fig. S7 for a schematic of the modification stages identified on the elephant-bone tool.

The broader context of the manufacture and use of the elephant bone knapping percussor from Boxgrove is greatly enhanced by the large-scale excavations at the site, combined with meticulous analysis of the knapping features, and its burial context within fine-grained intertidal deposits and minimally disturbed archeological layers in this coastal mudflat environment. Together, these factors provide a rare and detailed insight into the percussor’s life history within its broader landscape context (Fig. 10), a level of understanding seldom achievable at most Lower Paleolithic sites.

The rarity of elephant bones in the extensive area of the coastal plain excavated and sampled at Boxgrove suggests that the elephant bone used to make the knapping tool was sourced from beyond the immediate vicinity. Regardless of the cause of death—predation, disease, or another natural cause—the bones would have been scattered as the carcass decayed and subjected to weathering and shattering by various processes (*93*–*95*). These processes could have included trampling from elephants visiting the carcass (*96*) and trampling by other megafauna, as well as the actions of scavengers (*97*) and humans working on the carcass as a source of food and raw materials.

Any of the substantial limb bones would have provided a source of usable fragments (*98*), either by intentional smashing with a large boulder (*95*) or by picking up a naturally fractured piece from the carcass site. Although the fracture characteristics and knapping damage are consistent with those typically observed in fresh (green) bone, determining the exact time elapsed between the elephant’s death and the exploitation and working of the blank is difficult. While the source and nature of the elephant bone fragment remain elusive, the next phase involved intentional flaking using a handheld hammer to shape the piece and reduce its size to a manageable dimension, suitable for palmheld use. This process would have produced cortical flakes and bone chips. The flake scars indicate that at least three blows were aimed at opposing ends of the piece removing large cortical flakes, as well as marginal chipping along one of the edges that would have produced smaller, irregular bone fragments. The absence of such pieces in the excavated trench suggests that this preparatory flaking stage also occurred elsewhere in the landscape before the knapping tool was transported to the site where it was used to work flint.

Features of the knapping damage suggest that the percussor was used in relatively light knapping tasks, likely to maintain a sharp edge on a cutting tool that had become blunt during butchery. This process would have produced small flint chips, mostly less than 10 mm in length, based on the size of the knapping marks. These flint chips consistently appear in sieved samples of the intertidal deposit from sample locations across both quarries, indicating a background signature of resharpening events. This “background” scatter of small flint chips is dispersed between localized areas with dense clusters of knapping debris, discarded stone tools, and butchered large mammal bones found at several levels within the mudflat succession (*54*). The importance of this observation lies in recognizing that the zones between these high-intensity activity areas are not archeologically barren; instead, they contain subtle evidence of hominin activity in the form of flint tool resharpening events. These resharpening activities are typically the only trace of hominin presence in most of the excavated areas of the mudflats. These resharpening events are almost certainly linked to butchery episodes, as indicated by the presence of a low density of large mammal bones in this deposit, most of which bear cut marks or impact marks from marrow extraction (*53*).

The elephant bone percussor was a curated tool that was used on multiple occasions. Its “disposal” at the Q1/B mudflat location may have been accidental, or it may have been deliberately left behind for later use and not recovered. Alternatively, it could have been surplus to immediate requirements or abandoned for logistical reasons, with an individual or the group choosing to leave objects behind rather than carry them away from an activity area, such as a butchery site, to prioritize carrying other items, such as food or raw materials, and optimize their load or travel efficiency.

### Boxgrove within the Acheulean knapping traditions and percussion technology

The Acheulean handaxe technology, which Lycett and Gowlett (*99*) described as “the longest-lasting entity in the human cultural record,” originated in eastern Africa about 1.95 Ma (*100*, *101*). It then spread across other parts of Africa and into much of western and southern Asia, as well as Europe after about 1.5 Ma (*102*–*109*), possibly persisting until around 200,000 to 100,000 years ago [(*110*), but see (*111*)]. The hallmark tools of this tradition are handaxes, cleavers, and other large cutting tools, flaked on both sides to create a sharp cutting edge, often extending around the entire perimeter. The earliest handaxes in Africa coincide with the presence of *Homo ergaster* and *Homo erectus*, and during the expansion phase with later populations, including *Homo heidelbergensis* and early Neanderthals (*110*). According to some archeologists [e.g., (*112*)], once established, the Acheulean industry exhibited conservatism, persisting largely unchanged across extensive temporal and geographical spans. Despite this apparent consistency, however, Early and Late Acheulean handaxes [as defined by (*101*, *104*)] exhibit important differences.

The chronology of the Early and Late Acheulean lacks precise dates for many sites and regions (*100*, *101*, *104*, *113*). However, current evidence suggests that these phases represent two periods of relative stability, separated by a “step change” toward the end of the Early Pleistocene–early Middle Pleistocene in Africa that resulted in more refined handaxes and possibly coincided with the emergence and expansion of previously unknown hominin species (*110*, *114*).

Morphologically, Early Acheulean handaxes are thicker and less extensively trimmed and shaped with a lower degree of symmetry than Late Acheulean handaxes. They typically show fewer flake removals with larger and deeper flake scars and a relatively simple reduction strategy (*71*, *115*, *116*). Late Acheulean handaxes, in contrast, are typically highly symmetrical not just in plan form but also when viewed edge on. They are also characterized by extensive thinning associated with shallower and flatter flake scars, resulting from the final shaping and thinning stage. At Boxgrove, the waste flakes that are the by-products of this final shaping and thinning stages in the production of handaxes exhibit carefully prepared platforms (*71*), which are consistent with the development of the more technically challenging knapping techniques required to produce thinner and highly symmetrical handaxes (*71*, *116*).

One region that has recently received attention for studying the transition from Early Acheulean to the Late Acheulean is the chalklands of southern England (*115*, *117*–*124*). These studies have their origins in Derek Roe’s pioneering metrical analysis of British handaxes in the 1960s and 1970s (*121*, *122*). Roe identified a clear division between two sets of handaxes. The first (group V), currently assigned in part to MIS 15 (663,000 to 621,000 years ago), consists of “crude” handaxes with minimal flaking, thick (bifacial, trihedral, or irregular) profiles, and zigzag edges, which were knapped with a hard hammer. The second set includes a group of finely made elongated ovate handaxes with straight edges (group VII), to which the Boxgrove handaxes can now be added. Handaxes in this group, which are now assigned to MIS 13 (524,000 to 478,000 years ago), were finished using the soft hammer technique. Handaxes in groups V and VII represent the earliest handaxe industries in Britain, where later Acheulean occupations occurred during successive temperate stages, each characterized by distinct styles of handaxe types (*117*–*119*, *123*, *124*).

The transition from Early and Late Acheulean handaxes carries far-reaching behavioral and evolutionary implications, suggesting that they may have been made by distinct hominin groups with distinct technological and cognitive capabilities. In the Early Acheulean, a simpler knapping strategy was used by hominins lacking the cognitive ability or technological knowledge to procure and use soft hammers. By contrast, the production of Late Acheulean handaxes, including the Boxgrove handaxes, involved a demonstrably more complex process, integrating careful procurement and testing of raw material, deliberate platform preparation, and the selection of use-specific soft hammers (made of antler or bone) in the later stages of the lithic reduction sequence. These techniques were used to shape and thin the handaxes, as well as to create tranchet cutting tips (*60*–*62*, *73*).

The selection of suitable raw materials, and their modification to make usable soft hammers from giant deer and red deer antlers and various limb bones of megafauna, including the elephant bone knapping hammer described here, is one of the more notable aspects of the Boxgrove archeological record (*60*–*62*, *73*). The Boxgrove osseous artifact assemblage includes two carefully worked and heavily used antler knapping hammers for which no comparable artifacts are known in Europe until the Aurignacian <43,000 years ago or later (*91*, *125*). Before the discovery of a varied bone industry at Boxgrove (*60*, *62*), the prevailing belief was that “only Upper Paleolithic and Later Stone Age populations routinely turned bone and related substances into unequivocal artifacts within a curated technological framework” (*126*). Lower and Middle Paleolithic osseous tools were characterized as simple tools selected from readily available objects with minimal planning or preparation, serving immediate needs. They typically consisted of long-bone shaft fragments from butchery waste, often with minimal surface scraping preparation, reflecting their ad hoc nature (*127*, *128*). These tools were primarily used for specific shaping or resharpening tasks and were often discarded on the spot, rather than retained or extensively reused. In contrast, the antler knapping hammers from Boxgrove exemplify a more sophisticated approach to the production and use of knapping tools. They represent a curated technology, reflecting deliberate and intentional choices in the selection of raw materials and their subsequent production and use as specialized knapping tools. Unlike ad hoc tools, the antler hammers were intensively used and transported from location to location, where they were used to shape and resharpen a large number of handaxes over time. Moreover, the range of bones used as knapping tools, along with the variability in the types of knapping damage observed, suggests a variety of functional uses at different stages of the reduction and reshaping sequence.

An unexpected result from the analysis of the knapping tools at the Horse Butchery Site (Q2 GTP 17) at Boxgrove was the recognition that curation of knapping tools extended beyond antler knapping hammers. At least one of the bones used to work the flints at this site were identified as previously shaped pieces brought to the butchery site from other locations in the landscape (*61*, *62*). The other knapping tools from the Horse Butchery Site, however, better fit the category of ad hoc tools. These include broken bone splinters, including pieces derived from the butchered horse, which were used with minimal modification to resharpen the edges of flint tools before being discarded on-site (*61*, *62*). The elephant-bone retoucher is another example of a transported and curated knapping tool, in this case shaped by flaking a thick cortical elephant bone, a material seemingly rare in the Boxgrove landscape.

The spatial distribution of handaxes and knapping waste at Boxgrove, as well as the various types of knapping percussors, provides additional information about how early Middle Pleistocene hominins operated in the landscape. For example, the knapping “toolkit” included various cortical flint cobbles and beach pebbles used as hard hammers for roughing out handaxe blanks that mostly took place at the flint source near the cliff; large bone and antler soft hammers for heavy percussion to facilitate shaping and finishing; and smaller bone fragments for resharpening and reconfiguring lithic tool edges. It was the use of this combination of different knapping tools that facilitated the production of the elegantly and expertly shaped ovate handaxes, skillfully sharpened by the removal of tranchet flakes, which are the hallmarks of the Boxgrove lithic technology.

The variety of specifically selected knapping tools used by the Boxgrove flint knappers provides a link between the increase in toolmaking complexity seen in these late Acheulean lithic tools with a level of technological foresight and logistical planning. The use of both curated and ad hoc knapping tools when circumstances dictated reflects a highly flexible and adaptable approach to tasks and problem-solving in this early Middle Pleistocene population.

Boxgrove is unusual among Lower Paleolithic sites in the abundance and diversity of bone and antler knapping tools. The Boxgrove knapping toolkit includes rare evidence for the early adoption of antler knapping hammers, more commonly associated with Upper Paleolithic knapping technologies and modern experimental flintknappers. At Boxgrove, soft hammers were used in the early stages in the production of handaxes, with large shaping flakes removed from the rough-out with considerable force at manufacturing sites. In addition, soft hammers were used for lighter tasks, such as reshaping or refining cutting edges at butchery sites. The elephant-bone “retoucher” is an example of the latter type. The adoption of this combination of soft-hammer types and their varied uses represented a key technological development, facilitating the standardization of the symmetrical ovate shape and size observed in the Boxgrove handaxes. It is plausible to speculate that such handaxes not only served as more efficient cutting and butchery tools but also could have been more easily transported from site to site.

### Implications

The modification of an elephant bone into a knapping tool sheds light on another aspect of early Middle Pleistocene hominin behavior: The deliberate selection of a particularly thick-walled and durable cortical bone material of a type that was infrequently encountered within the local environment. This find provides further evidence of the strategic selection and curation of organic tools among early hominins, implying a high level of resourcefulness, adaptability, and a nuanced understanding of their environment and available materials. These strategies are facets of an increasingly complex set of behaviors and a flexible technological repertoire that enabled them to carve out new niches during the northern expansion of archaic human populations. It was this adaptability that served as one of the critical factors enabling them to thrive in a broader range of cooler and seasonally variable environments encountered in early Middle Pleistocene Europe, approximately 500 ka.

## MATERIALS AND METHODS

The elephant bone NHMUK PV UNREG 4339 was examined using a binocular microscope (Nikon SMZ-10) with up to ×40 magnification and fiber-optic illumination. The microscope’s camera lucida was used to make drawings of features of interest, which were subsequently superimposed on annotated photographs of the knapping area. Photographic images were captured using reflectance transformation imaging, a digital microscope (Dino-Lite Edge AM73915MZTL USB 3.0 digital microscope, up to ×140 magnification) and an SEM (the JEOL IT500 SEM) operated in variable pressure mode (chamber pressure ~ 70 Pa) with elemental mapping. 3D models of the specimen were captured with an Artec Space Spider (e.g., fig. S1B), and high-resolution 3D imaging of surface features was undertaken with a focus variation microscope (Alicona InfiniteFocus G5+) optical surface measurement system. To identify flint chips (primarily composed of silicon dioxide) embedded in the surface of bone (calcium-rich hydroxyapatite) during the knapping process, we coupled SEM analysis with an EDX detector to map the distribution of calcium and silicon. Additional imaging was undertaken with a TOMLOV digital microscope (figs. S3 to S5)

## Supplementary Materials

### This PDF file includes:

Figs. S1 to S7 Tables S1 and S2 References

## References

[1] A. M. Lister, A. J. Stuart, The West Runton mammoth (*Mammuthus trogontherii*) and its evolutionary significance. Quat. Internat. 228, 80–209 (2010).

[2] E. E. Erkek, A. M. Lister, The skeleton of a straight-tusked elephant, *Palaeoloxodon antiquus* (Falconer and Cautley, 1847) from Selsey, England, and growth and variation in *Palaeoloxodon* of the European Pleistocene. J. Quat. Sci. 36, 211–223 (2021).

[3] A. J. Stuart, *Vanished Giants. The Lost World of the Ice Age* (The University of Chicago Press, 2021).

[4] T. Hauffe, J. L. Cantalapiedra, D. Silvestro, Trait-mediated speciation and human-driven extinctions in proboscideans revealed by unsupervised Bayesian neural networks. Sci. Adv. 10, eadl2643 (2024). 39047110 10.1126/sciadv.adl2643 PMC11268411

[5] G. M. Bhat, N. Ashton, S. Parfitt, A. Jukar, M. R. Dickinson, B. Thusu, J. Craig, Human exploitation of a straight-tusked elephant (*Palaeoloxodon*) in Middle Pleistocene deposits at Pampore, Kashmir, India. Quat. Sci. Rev. 342, 108894 (2024).

[6] S. Gaudzinski-Windheuser, L. Kindler, K. MacDonald, W. Roebroeks, Hunting and processing of straight-tusked elephants 125.000 years ago: Implications for Neanderthal behavior. Sci. Adv. 9, eadd8186 (2023). 36724231 10.1126/sciadv.add8186 PMC9891704

[7] S. Gaudzinski-Windheuser, L. Kindler, W. Roebroeks, Widespread evidence for elephant exploitation by Last Interglacial Neanderthals on the North European plain. Proc. Natl. Acad. Sci. U.S.A. 120, e2309427120 (2023). 38048457 10.1073/pnas.2309427120 PMC10723128

[8] S. Gaudzinski, E. Turner, A. P. Anzidei, E. Alvarez-Fernández, J. Arroyo-Cabrales, J. Cinq-Mars, V. T. Dobosi, A. Hannus, E. Johnson, S. C. Munzel, A. Scheer, P. Villa, The use of proboscidean remains in every-day Palaeolithic life. Quat. Int. 126-128, 179–194 (2005).

[9] G. Boschian, D. Saccà, In the elephant, everything is good: Carcass use and re-use at Castel di Guido (Italy). Quat. Int. 361, 288–296 (2015).

[10] L. Buck, C. Stringer, Having the stomach for it: A contribution to Neanderthal diets? Quat. Sci. Rev. 96, 161–167 (2014).

[11] G. E. Konidaris, R. Barkai, V. Tourloukis, K. Harvati, Eds., *Human-Elephant Interactions: From Past To Present* (Tübingen Univ. Press, 2021).

[12] G. Boschian, D. Caramella, D. Saccà, R. Barkai, Are there marrow cavities in Pleistocene elephant limb bones, and was marrow available to early humans? New CT scan results from the site of Castel di Guido (Italy). Quat. Sci. Rev. 215, 86–97 (2019).

[13] A. Lister, P. G. Bahn, *Mammoths: Giants of the Ice Age* (University of California Press, 2007).

[14] N. J. Conard, M. Malina, S. C. Münzel, New flutes document the earliest musical tradition in southwestern Germany. Nature 460, 737–740 (2009). 19553935 10.1038/nature08169

[15] P. Valde-Nowak, A. Nadachowski, M. Wolsan, Upper Palaeolithic boomerang made of a mammoth tusk in south Poland. Nature 329, 436–438 (1987).

[16] N. J. Conard, Palaeolithic ivory sculptures from southwestern Germany and the origins of figurative art. Nature 426, 830–832 (2003). 14685236 10.1038/nature02186

[17] C.-J. Kind, N. Ebinger-Rist, S. Wolf, T. Beutelspacher, K. Wehrberger, The smile of the Lion Man. Recent excavations in Stadel Cave (Baden-Württemberg, southwestern Germany) and the restoration of the famous Upper Palaeolithic figurine. Quartär 61, 129–145 (2014).

[18] M. Oliva, “The Brno II Upper Palaeolithic burial,” in *Hunters of the Golden Age*, W. Roebroeks, M. Mussi, J. Svoboda, K. Fennema, Eds. (Faculty of Archaeology, 2000), pp. 143–159.

[19] M. Sablin, N. Reynolds, K. Iltsevich, M. Germonpréc, The Epigravettian site of Yudinovo, Russia: Mammoth bone structures as ritualised middens. Environ. Archaeol. 30, 1–21 (2023).

[20] O. Soffer, *The Upper Paleolithic of the Central Russian Plain* (Academic Press, 1985).

[21] R. G. Klein, *Ice-Age Hunters of the Ukraine* (The University of Chicago Press, 1973).

[22] P. Callow, J. M. Cornford, Eds., *La Cotte de St Brelade 1961–1978. Excavations by C.B.M. McBurney* (Geobooks, 1986).

[23] K. Scott, Two hunting episodes of Middle Palaeolithic age at La Cotte de Saint-Brelade, (Channel Islands), Jersey. World Archaeol. 12, 137–152 (1980).

[24] K. Scott, “The bone assemblages from layers 3 and 6,” in *La Cotte de St Brelade 1961–1978. Excavations by C.B.M. McBurney*, P. Callow, J. M. Cornford, Eds. (Geobooks, 1986), pp. 159–185.

[25] B. Scott, M. Bates, R. Bates, C. Conneller, M. Pope, A. Shaw, G. Smith, A new view from La Cotte de St Brelade, Jersey. Antiquity 88, 13–29 (2014).

[26] B. Scott, A. Shaw, K. Scott, M. Pope, *Repeopling La Manche: New Perspectives on Neanderthal Lifeways from La Cotte de St Brelade*, Prehistoric Society Research Paper 10 (The Prehistoric Society, 2023).

[27] A. Shaw, B. Scott, M. Pope, “The early Middle Palaoelithic ‘bone heaps’ from La Cotte de St Brelade reconsidered,” in *Repeopling La Manche: New Perspectives on Neanderthal Lifeways from La Cotte de St Brelade*, B. Scott, A. Shaw, K. Scott, M. Pope, Eds., Prehistoric Society Research Paper 10 (The Prehistoric Society, 2022), pp. 43–64.

[28] C. B. Stringer, *Homo Britannicus: The Incredible Story of Human Life in Britain*. (Penguin Books Ltd., 2007), vol. 27, pp. 39–40.

[29] F. Wenban-Smith, “The essential elephant: Northwest European Hominin adaptations through the Middle-Late Pleistocene and Neanderthal extinction,” in *Human-Elephant Interactions from Past to Present*, G. E. Konidaris, R. Barkai, V. Tourloukis, K. Harvati, Eds. (Tübingen Univ. Press, Tübingen, 2021), pp. 145–176.

[30] M. D. Leakey, *Olduvai Gorge: Excavations in Bed I and II, 1960–1963* (Cambridge Univ. Press, 1971).

[31] P. Shipman, “Altered bones from Olduvai Gorge, Tanzania: Techniques, problems and implications for their recognition,” in *Bone Modification*, R. Bonnichsen, M. H. Sorg, Eds. (University of Maine Centre for the Study of the First Americans, Thompson-Shore Inc., 1989), pp. 317–334.

[32] L. R. Backwell, F. d’Errico, The first use of bone tools: A reappraisal of the evidence from Olduvai Gorge, Tanzania. Palaeontol. Afr. 40, 95–158 (2004).

[33] L. Backwell, F. d’Errico, “The origin of bone tool technology and the identification of early hominid cultural traditions,” in *From Tools to Symbols: From Early Hominids to Modern Humans*, F. d’Errico, L. Backwell, Eds. (Wits Univ. Press, 2005), pp. 238–275.

[34] L. R. Backwell, F. d’Errico, “Palaeolithic bone tools,” in *Encyclopedia of Global Archaeology*, C. Smith, Ed. (Springer, 2014), pp. 950–962.

[35] M. Pante, I. de la Torre, F. d’Errico, J. Njau, R. Blumenschine, Bone tools from Beds II-IV, Olduvai Gorge, Tanzania, and implications for the origins and evolution of bone technology. J. Hum. Evol. 148, 102885 (2020). 33049586 10.1016/j.jhevol.2020.102885

[36] I. de la Torre, L. Doyon, A. Benito-Calvo, R. Mora, I. Mwakyoma, J. K. Njau, R. F. Peters, A. Theodoropoulou, F. d’Errico, Systematic bone tool production at 1.5 million years ago. Nature 640, 130–134 (2025). 40044851 10.1038/s41586-025-08652-5 PMC11964934

[37] F. Marinelli, M.-H. Moncel, C. Lemorini, The use of bones as tools in Late Lower Paleolithic of Central Italy. Sci. Rep. 14, 11666 (2024). 38778167 10.1038/s41598-024-62612-z PMC11111801

[38] K. Zutovski, R. Barkai, The use of elephant bones for making Acheulian handaxes: A fresh look at old bones. Quat. Int. 406, 227–238 (2016).

[39] R. Barkai, The elephant in the handaxe: Lower Palaeolithic ontologies and representations. Camb. Archaeol. J. 31, 349–361 (2021).

[40] I. Biddittu, P. Celletti, “Plio-Pleistocene Proboscidea and Lower Palaeolithic bone industry of southern Latium (Italy),” in *The World of Elephants* (International Congress, 2001), pp. 91–96.

[41] A. G. Costa, “A geometric morphometric assessment of plan shape in bone and stone Acheulean bifaces from the Middle Pleistocene site of Castel di Guido, Latium, Italy,” in *New Perspectives on Old Stones*, S. Lycett, P. Chauhan, Eds. (Springer, 2010), pp. 23–41.

[42] S. A. Semenov, *Prehistoric Technology: An Experimental Study of the Oldest Tools and Artefacts from Traces of Manufacture and Wear*, M. W. Thompson, Transl. (Cory, Adams & Mackay, London, 1964).

[43] G. Haynes, *Mammoths, Mastodonts, and Elephants: Biology, Behavior, and the Fossil Record* (Cambridge Univ. Press, 1991).

[44] J. M. Hutson, A. García-Moreno, E. S. Noack, E. Turner, A. Villaluenga, S. Gaudzinski-Windheuser, “The origins of bone tool technologies: Conclusions and future directions,” in *The Origins of Bone Tool Technologies*, J. M. Hutson, A. García-Moreno, E. S. Noack, E. Turner, A. Villaluenga, S. Gaudzinski-Windheuser, Eds. (RGZM, 2018), pp. 317–326.

[45] M. D. Leakey, D. A. Roe, *Olduvai Gorge 5: Excavations in Beds III, IV and the Masek Beds, 1968–1971* (Cambridge Univ. Press, 1994).

[46] J. K. Njau, R. J. Blumenschine, A diagnosis of crocodile feeding traces on larger mammal bone, with fossil examples from the Plio-Pleistocene Olduvai Basin, Tanzania. J. Hum. Evol. 50, 142–162 (2006). 16263152 10.1016/j.jhevol.2005.08.008

[47] J. K. Njau, H. G. Gilbert, “Standardizing terms for crocodile-induced bite marks on bone surfaces in light of the frequent bone modification equifinality found to result from crocodile feeding behavior, stone tool modification, and trampling,” in *FOROST Occasional Publication* (2016), vol. 3, pp. 1–13.

[48] Y. Sahle, S. El Zaatari, T. D. White, Hominid butchers and biting crocodiles in the African Plio-Pleistocene. Proc. Natl. Acad. Sci. U.S.A. 114, 13164–13169 (2017). 29109249 10.1073/pnas.1716317114 PMC5740633

[49] V. Tourloukis, N. Thompson, E. Panagopoulou, D. Giusti, G. E. Konidaris, P. Karkanas, K. Harvatia, Lithic artifacts and bone tools from the Lower Palaeolithic site Marathousa 1, Megalopolis, Greece: Preliminary results. Quat. Int. 497, 47–64 (2018).

[50] S. M. Bello, S. A. Parfitt, Taphonomic approaches to distinguish chewing damage from knapping marks in Palaeolithic faunal assemblages. J. Archaeol. Sci. Rep. 51, 104183 (2023).

[51] A. Woodcock, *The Lower and Middle Palaeolithic of Sussex*, B.A.R British Series 94 (BAR Publishing, 1981).

[52] M. B. Roberts, S. A. Parfitt, M. I. Pope, F. F. Wenban-Smith, R. I. Macphail, A. Locker, J. R. Stewart, Boxgrove, West Sussex: Rescue excavations of a Lower Palaeolithic landsurface (Boxgrove Project B, 1989–1991). Proc. Prehist. Soc. 63, 303–358 (1997).

[53] M. B. Roberts, S. A. Parfitt, *Boxgrove. A Middle Pleistocene Hominid Site at Eartham Quarry, Boxgrove, West Sussex*, English Heritage Archaeological Report 17 (English Heritage, 1999).

[54] M. Pope, S. Parfitt, M. Roberts, *The Horse Butchery Site: A High-Resolution Record of Lower Palaeolithic Hominin Behaviour at Boxgrove, UK* (SpoilHeap Publications, 2020).

[55] R. C. Preece, S. A. Parfitt, Environmental heterogeneity of the Lower Palaeolithic land surface on the Goodwood-Slindon Raised Beach: Comparisons of the records from Boxgrove and Valdoe, Sussex, UK. J. Quat. Sci. 37, 572–592 (2022).

[56] S. A. Parfitt, R. C. Preece, New palaeontological evidence suggests an early Middle Pleistocene age for the lower levels of Sun Hole Cave, Cheddar, Somerset, UK. Proc. Geol. Assoc. 133, 162–175 (2022).

[57] C. B. Stringer, E. Trinkaus, M. B. Roberts, S. A. Parfitt, R. I. Macphail, The Middle Pleistocene human tibia from Boxgrove. J. Hum. Evol. 34, 509–547 (1998). 9614636 10.1006/jhev.1998.0215

[58] S. Hillson, S. A. Parfitt, S. M. Bello, M. B. Roberts, C. B. Stringer, Two hominin incisor teeth from the Middle Pleistocene site of Boxgrove, Sussex, England. J. Hum. Evol. 59, 493–503 (2010). 20828787 10.1016/j.jhevol.2010.06.004

[59] A. L. Lockey, L. Rodríguez, L. Martín-Francés, J. L. Arsuaga, J. M. Bermúdez de Castro, L. Crété, M. Martinón-Torres, S. Parfitt, M. Pope, C. Stringer, Comparing the Boxgrove and Atapuerca (Sima de los Huesos) human fossils: Do they represent distinct paleodemes? J. Hum.Evol. 172, 10325 (2022). 10.1016/j.jhevol.2022.103253 36162354

[60] M. W. Pitts, M. Roberts, *Fairweather Eden: Life in Britain Half a Million Years Ago as Revealed by the Excavations at Boxgrove* (Century, 1997).

[61] S. A. Parfitt, S. M. Bello, “The manufacture and use of bone tools,” in *The Horse Butchery Site: A High-resolution Record of Lower Palaeolithic Hominin Behaviour at Boxgrove, UK*, M. Pope, S. Parfitt, M. Roberts, Eds. (SpoilHeap Publications, 2020), pp. 105–121.

[62] S. A. Parfitt, S. M. Bello, Bone tools, carnivore chewing and heavy percussion: Assessing conflicting interpretations of Lower and Upper Palaeolithic bone assemblages. R. Soc. Open Sci. 11, 11231163 (2024). 10.1098/rsos.231163 PMC10762443 38179084

[63] S. A. Parfitt, M. D. Lewis, S. M. Bello, Taphonomic and technological analyses of Lower Palaeolithic bone tools from Clacton-on-Sea, UK. Sci. Rep. 12, 20222 (2022). 36418870 10.1038/s41598-022-23989-x PMC9684524

[64] L. Sánchez-Romero, A. Benito-Calvo, D. De Loecker, M. Pope, Spatial analysis and site formation processes associated with the Middle Pleistocene hominid teeth from Q1/B waterhole, Boxgrove (West Sussex, UK). Archaeol. Anthropol. Sci. 15, 98 (2023).

[65] M. Pope, “*The Significance of Biface-rich Assemblages: An Examination of Behavioural Control on Lithic Assemblage Formation in the Lower Palaeolithic*”, thesis, University of Southampton, UK (2001).

[66] P. García-Medrano, A. Ollé, N. Ashton, M. B. Roberts, The mental template in handaxe manufacture: New insights into Acheulean lithic technological behavior at Boxgrove, Sussex, UK. J. Archaeol. Method Theory 26, 396–422 (2019).

[67] J. C. Mitchell, “A use-wear analysis of selected British Lower Palaeolithic handaxes with special reference to the site of Boxgrove (West Sussex): A study incorporating optical microscopy, computer aided image analysis and experimental archaeology,” thesis, University of Oxford, UK (1998).

[68] R. Iovita, S. P. McPherron, The handaxe reloaded: A morphometric reassessment of Acheulian and Middle Paleolithic handaxes. J. Hum. Evol. 61, 61–74 (2011). 21496877 10.1016/j.jhevol.2011.02.007

[69] C. Shipton, F. Foulds, A. Rawlinson, M. Leroyer, N. Ashton, M. White, Turning-the-edge, tranchet, and social signalling at Boxgrove. Cam. Archaeol. J. 2025, 1–18 (2025).

[70] C. Shipton, C. Clarkson, R. Cobden, Were Acheulean bifaces deliberately made symmetrical? Archaeological and experimental evidence. Camb. Archaeol. J. 29, 65–79 (2019).

[71] F. F. Wenban-Smith, The use of canonical variates for determination of biface manufacturing technology at Boxgrove Lower Palaeolithic site and the behavioural implications of this technology. J. Archaeol. Sci. 16, 17–26 (1989).

[72] D. Stout, J. Apel, J. Commander, M. Roberts, Late Acheulean technology and cognition at Boxgrove, UK. J. Archaeol. Sci. 41, 576–590 (2014).

[73] M. B. Roberts, S. A. Parfitt, “The bone hammers from the early Middle Pleistocene site at Boxgrove, West Sussex, UK: Their identification, process of manufacturing, and use,” in *Retouching the Palaeolithic: Becoming Human and the Origins of Bone Tool Technology*, J. Hutson, A. García-Moreno, A. Villaluenga, Eds. (MONREPOS Research Centre and Museum for Human Behavioural Evolution – RGZM, 2015), p. 48.

[74] A. V. Neal, “The analysis and investigation of partially fossilized bone material from the Lower Palaeolithic site at Amey’s Eartham Pit, Boxgrove, West Sussex”, thesis, University of London, UK (1987).

[75] M. M. E. Jans, “Microscopic destruction of bone,” in *Manual of Forensic Taphonomy*, J. T. Pokines, E. N. L’Abbé, S. A. Symes, Eds. (CRC Press, 2021), pp. 23–40.

[76] M. M. E. Jans, C. M. Nielsen-Marsh, C. I. Smith, M. J. Collins, H. Kars, Characterisation of microbial attack on archaeological bone. J. Archaeol. Sci. 31, 87–95 (2004).

[77] C. J. Hackett, Microscopical focal destruction (tunnels) in exhumed human bones. Med. Sci. Law 21, 243–265 (1981). 7321807 10.1177/002580248102100403

[78] V. Marchiafava, E. Bonucci, A. Ascenzi, Fungal osteoclasia: A model of dead bone resorption. Calcif. Tissue Int. 14, 195–210 (1974). 10.1007/BF02060295 4843788

[79] Y. Fernández-Jalvo, P. Andrews, D. Pesquero, C. Smith, D. Marín-Monfort, B. Sánchez, E.-M. Geigl, A. Alonso, Early bone diagenesis in temperate environments. Part I: Surface features and histology. Palaeogeogr. Palaeoclimatol. Palaeoecol. 288, 62–81 (2010).

[80] G. Turner-Walker, Light at the end of the tunnels? The origins of microbial bioerosion in mineralised collagen. Palaeogeogr. Palaeoclimatol. Palaeoecol. 529, 24–38 (2019).

[81] A. M. Lister, The stratigraphical significance of deer species in the Cromer Forest-bed Formation. J. Quat. Sci. 8, 95–108 (1993).

[82] A. M. Lister, “Ecological Interactions of Elephantids in Pleistocene Eurasia: *Palaeoloxodon* and *Mammuthus*,” in *Human Paleoecology in the Levantine Corridor*, N. Goren-Inbar, J. D. Speth, Eds. (Oxbow Books, 2017), pp. 53–60.

[83] A. M. Lister, Dating the arrival of straight-tusked elephant (*Palaeoloxodon* spp.) in Eurasia. *Bulletin Du Musée d’anthropologie Préhistorique De Monaco* Supplément 6, 123–128 (2016).

[84] R. Potts, P. Shipman, Cutmarks made by stone tools on bones from Olduvai Gorge, Tanzania. Nature 291, 577–580 (1981).

[85] Y. Fernández-Jalvo, P. Andrews, *Atlas of Taphonomic Identifications. 1001+ Images of Fossil and Recent Mammal Bone Modification* (Springer, 2016).

[86] T. van Kolfschoten, S. A. Parfitt, J. Serangeli, S. M. Bello, Lower Paleolithic bone tools from the “Spear Horizon” at Schöningen (Germany). J. Hum. Evol. 89, 226–263 (2015). 26653208 10.1016/j.jhevol.2015.09.012

[87] J. M. Hutson, A. García-Moreno, E. S. Noack, E. Turner, A. Villaluenga, S. Gaudzinski-Windheuser, Eds., *The Origins of Bone Tool Technologies* (RGZM, 2018).

[88] M. É. Dupont, *Les Temps Antéhistoriques en Belgique: L’Homme Pendant les Âges de la Pierre dans les Environs de Dinant-sur-Meuse* (Muquardt, 1871).

[89] F. Daleau, “Sur des lésions que présentent certains os de la période paléolithique”, in *Association Française pour l’Avancement des Sciences, Compte Rendu de la 12e Session, Rouen 1883* (Chaix, 1884), pp. 600–602.

[90] M. Patou-Mathis, Ed., *Retouchoirs, Compresseurs, Percuteurs. Os à Impressions et à Éraillures. Fiches Typologiques de l’Industrie Osseuse Préhistorique, Cahier X* (Éditions Société Préhistorique Française, 2002).

[91] S. M. Bello, G. Delbarre, I. De Groote, S. A. Parfitt, A newly discovered antler flint-knapping hammer and the question of their rarity in the Palaeolithic archaeological record: Reality or bias? Quat. Int. 403, 107–117 (2016).

[92] J. B. Mallye, C. Thiébaut, V. Mourre, S. Costamagno, É. Claud, P. Weisbecker, The Mousterian bone retouchers of Noisetier Cave: Experimentation and identification of marks. J. Archaeol. Sci. 39, 1131–1142 (2012).

[93] A. K. Behrensmeyer, The bones of Amboseli: Bone assemblages and ecological change in a modern African ecosystem. Natl. Geogr. Res. 9, 402–421 (1993).

[94] G. Haynes, Longitudinal studies of African elephant death and bone deposits. J. Archaeol. Sci. 15, 131–157 (1988).

[95] G. Haynes, K. Krasinski, P. Wojtal, A study of fractured proboscidean bones in recent and fossil assemblages. J. Archaeol. Method Theory 28, 956–1025 (2021).

[96] L. Douglas-Hamilton, O. Doulas-Hamilton, *Among the Elephants* (The Viking Press, 1975).

[97] A. J. Stuart, N. Larkin, Taphonomy of the West Runton Mammoth. Quat. Int. 228, 217–232 (2010).

[98] P. Biberson, E. Aguirrre, Experiences de taille d’outils préhistoriques dans des os d’élephant. *Quaternaria*, 7, 165–183 (1965).

[99] S. J. Lycett, J. A. J. Gowlett, On questions surrounding the Acheulean ‘tradition’. World Archaeol. 40, 295–315 (2008).

[100] M. Mussi, J. Panera, S. Rubio-Jara, T. W. Davies, D. Geraads, H. Bocherens, G. Briatico, A. Le Cabec, J.-J. Hublin, A. Gidna, R. Bonnefille, L. Di Bianco, E. Méndez-Quintas, Early *Homo erectus* lived at high altitudes and produced both Oldowan and Acheulean tools. Science 382, 713–718 (2023). 10.1126/science.add9115 37824630

[101] R. G. Klein, The earlier stone age of Southern Africa. S. Afr. Archaeol. Bull. 55, 107–122 (2000).

[102] R. Klein, “Hominin dispersals in the Old World,” in *The Human Past. World Prehistory & the Development of Human Societies*, C. Scarre, Ed. (Thames & Hudson Ltd., ed. 3, 2013), pp. 84–123.

[103] O. Bar-Yosef, M. Belmaker, Early and Middle Pleistocene Faunal and hominins dispersals through Southwestern Asia. Quat. Sci. Rev. 30, 1318–1337 (2011).

[104] I. de la Torre, The origins of the Acheulean: Past and present perspectives on a major transition in human evolution. Phil. Trans. R. Soc. 371, 20150245 (2016). 10.1098/rstb.2015.0245 PMC4920301 27298475

[105] G. R. Scott, L. Gibert, The oldest hand-axes in Europe. Nature 461, 82–85 (2009). 19727198 10.1038/nature08214

[106] X. Li, H. Ao, M. J. Dekkers, A. P. Roberts, P. Zhang, S. Lin, W. Huang, Y. Hou, W. Zhang, Z. An, Early Pleistocene occurrence of Acheulian technology in North China. Quat. Sci. Rev. 156, 12–22 (2017).

[107] H. Li, K. Kuman, C. Li, What is currently (un)known about the Chinese Acheulean, with implications for hypotheses on the earlier dispersal of hominids. Comptes Rendus Palevol 17, 120–130 (2018).

[108] H. Yamei, R. Potts, Y. Baoyin, G. Zhengtang, A. Deino, W. Wei, J. Clark, X. Guangmao, H. Weiwen, Mid-Pleistocene Acheulean-like stone technology of the Bose Basin, South China. Science 287, 1622–1626 (2000). 10698732 10.1126/science.287.5458.1622

[109] S. Pappu, Y. Gunnell, K. Akhilesh, R. Braucher, M. Taieb, F. Demory, N. Thouveny, Early Pleistocene presence of Acheulian hominins in South India. Science 331, 1596–1599 (2011). 21436450 10.1126/science.1200183

[110] J. Galway-Witham, J. Cole, C. Stringer, Aspects of human physical and behavioural evolution during the last 1 million years. J. Quat. Sci. 34, 355–378 (2019).

[111] A. J. M. Key, I. Jarić, D. L. Roberts, Modelling the end of the Acheulean at global and continental levels suggests widespread persistence into the Middle Palaeolithic. Humanit. Soc. Sci. Commun. 8, 1–12 (2021).38617731

[112] J. Wymer, *The Palaeolithic Age* (Croom Helm, 1982).

[113] M. Mussi, E. Mendez-Quintas, D. Barboni, H. Bocherens, R. Bonnefille, G. Briatico, D. Geraads, R. T. Melis, J. Panera, L. Pioli, A. Serodio Domínguez, S. Rubio Jara, A surge in obsidian exploitation more than 1.2 million years ago at Simbiro III (Melka Kunture, Upper Awash, Ethiopia). Nat. Ecol. Evol. 7, 337–346 (2023). 36658266 10.1038/s41559-022-01970-1

[114] J. Paige, C. Perreault, 3.3 million years of stone tool complexity suggests that cumulative culture began during the Middle Pleistocene. Proc. Natl. Acad. Sci. U.S.A. 121, e2319175121 (2024). 38885385 10.1073/pnas.2319175121 PMC11214059

[115] D. A. Roe, Some Hampshire and Dorset handaxes and the question of ‘Early Acheulian’ in Britain. Proc. Prehist. Soc. 41, 1–9 (1975).

[116] J. Cook, R. Jacobi, “Observations on the Artefacts from the Breccia at Kent’s Cavern,” in *Stone Age Archaeology: Essays in Honour of John Wymer*, N. Ashton, F. Healy, P. Pettit, Eds., Oxbow Monograph 102, Lithics Study Society Occasional Paper 6 (Oxbow Books, 1998), pp. 77–89.

[117] M. J. White, D. R. Bridgland, D. C. Schreve, T. S. White, K. E. H. Penkman, Well-dated fluvial sequences as templates for patterns of handaxe distribution: Understanding the record of Acheulean activity in the Thames and its correlatives. Quat. Int. 480, 118–131 (2018).

[118] R. Davis, N. Ashton, M. Hatch, P. G. Hoare, S. G. Lewis, Palaeolithic archaeology of the Bytham River: Human occupation of Britain during the early Middle Pleistocene and its European context. J. Quat. Sci. 36, 526–546 (2021).

[119] P. García-Medrano, C. Shipton, M. White, N. Ashton, Acheulean diversity in Britain (MIS 15-MIS11): From the standardization to the regionalization of technology. Front. Earth Sci. 10, 917207 (2022).

[120] A. Key, T. Lauer, M. Skinner, M. Pope, D. R. Bridgland, L. Noble, T. Proffitt, On the earliest Acheulean in Britain: First dates and in-situ artefacts from the MIS 15 site of Fordwich (Kent, UK). R. Soc. Open Sci. 9, 211904 (2022). 35754990 10.1098/rsos.211904 PMC9214292

[121] D. A. Roe, British Lower and Middle Palaeolithic handaxe groups. Proc. Prehist. Soc. 34, 1–82 (1968).

[122] J. McNabb, Looking backwards, looking forwards: Evaluating the Roe handaxe methodology in the twenty-first century and the introduction of a new ‘Roe-type’ index. Lithic Technol. 47, 183–202 (2022).

[123] D. R. Bridgland, M. J. White, Fluvial archives as a framework for the Lower and Middle Palaeolithic: Patterns of British artefact distribution and potential chronological implications. Boreas 43, 543–555 (2014).

[124] D. R. Bridgland, M. J. White, Chronological variations in handaxes: Patterns detected from fluvial archives in north-west Europe. J. Quat. Sci. 30, 623–638 (2015).

[125] J. Orłowska, K. Cyrek, G. P. Kaczmarczyk, W. Migal, G. Osipowicz, Rediscovery of the Palaeolithic antler hammer from Biśnik Cave, Poland: New insights into its chronology, raw material, technology of production and function. Quat. Int. 665-666, 48–64 (2023).

[126] R. G. Klein, “Fully modern humans,” in *Archaeology at the Millennium*, G. M. Feinman, T. Douglas Price, Eds. (Springer, 2001), pp. 107–135.

[127] P. G. Chase, Tool-making tools and Middle Paleolithic behavior. Curr. Anthropol. 31, 443–447 (1990).

[128] J. Rosell, R. Blasco, J. F. Peris, E. Carbonell, R. Barkai, A. Gopher, Recycling bones in the Middle Pleistocene: Some reflections from Gran Dolina TD10-1 (Spain), Bolomor Cave (Spain) and Qesem Cave (Israel). Quat. Int. 361, 297–312 (2015).

[129] S. Lehnig, J. M. Hutson, E. Turner, A. Villaluenga, A. García-Moreno, G. Carver, S. Gaudzinski-Windheuser, Interpreting the Schöningen 13II-4 butchery sequence using the Harris Matrix. J. Archaeol. Sci. Rep. 36, 102833 (2021).

[130] P. Villa, G. Boschian, L. Pollarolo, D. Saccà, F. Marra, S. Nomade, A. Pereira, Elephant bones for the Middle Pleistocene toolmaker. PLOS ONE 16, e0256090 (2021). 34437571 10.1371/journal.pone.0256090 PMC8389514

[131] P. F. Cassoli, C. De Giuli, A. M. Radmilli, A. G. Segre, “Giacimento del Paleolitico inferiore a Malagrotta (Roma),” in *Atti XXIII Riunione Scientifica Istituto Italiano di Preistoria e Protostoria, Il Paleolitico inferiore in Italia, Firenze*, (Istituto Italiano di Preistoria e Protostoria, 1982), vol. 1982, pp. 531–549.

[132] R. Rabinovich, O. Ackermann, E. Aladjem, R. Barkai, R. Biton, I. Milevski, N. Solodenko, O. Marder, Elephants at the middle Pleistocene Acheulian open-air site of Revadim Quarry, Israel. Quat. Int. 276-277, 183–197 (2012).

[133] G. Gvirtzman, M. Wieder, O. Marder, H. Khalaily, R. Rabinovich, H. Ron, Geological and pedological aspects of an Early-Paleolithic site: Revadim, Central Coastal Plain, Israel. Geoarchaeology 14, 101–126 (1999).

[134] M. H. Moncel, I. Biddittu, G. Manzi, B. Saracino, A. Pereira, S. Nomade, C. Hertler, P. Voinchet, J.-J. Bahain, Emergence of regional cultural traditions during the Lower Palaeolithic: The case of Frosinone-Ceprano basin (Central Italy) at the MIS 11-10 transition. Archaeol. Anthropol. Sci. 12, 185 (2020).

[135] I. Biddittu, A. G. Segre, “Utilizzazione dell’osso nel Paleolitico inferiore italiano.” in *Atti XXIII Riunione Scientifica Istituto Italiano di Preistoria e Protostoria*, (Istituto Italiano di Preistoriae Protostoria, 1982), pp. 89–105.

[136] A. Ascenzi, *Dives Anagnia: archeologia nella valle del Sacco* (L’Erma di Bretschneider, 1993), pp. 38–47.

[137] A. Pereira, S. Nomade, M. H. Moncel, P. Voinchet, J. J. Bahain, I. Biddittu, C. Falguères, B. Giaccio, G. Manzi, F. Parenti, G. Scardia, V. Scao, G. Sottili, A. Vietti, Integrated geochronology of Acheulian sites from the southern Latium (central Italy): Insights on human-environment interaction and the technological innovations during the MIS 11-MIS 10 period. Quat. Sci. Rev. 187, 112–129 (2018).

[138] A. M. Rodmilli, G. Boschian, *Gli scavi a Castel di Guido. Il più antico giacimento di cacciatori nell’Agro Romano* (ETS, 1996).

[139] B. Mecozzi, I. Fiore, B. Giaccio, F. Giustini, S. Mercurio, L. Monaco, A. Argento, F. Bucci Casari degli Atti Di Sassoferrato, I. Caricola, C. Lemorini, F. Lucchini, I. Mazzini, M. R. Palombo, R. Sardella, A. Sposato, E. L. Spinapolice, F. Alhaique, From meat to raw material: The Middle Pleistocene elephant butchery site of Casal Lumbroso (Rome, central Italy). PLOS ONE 20, e0328840 (2025). 41060944 10.1371/journal.pone.0328840 PMC12507280

[140] D. Mania, U. Mania, “Bilzingsleben - *Homo erectus*, his culture and his environment. The most important results of research,” in *Lower Palaeolithic Small Tools in Europe and The Levant*, J. M. Burdukiewicz, A. Ronen, Eds., B.A.R British Series (BAR Publishing, 2003), pp. 29–48.

[141] E. Brühl, “The small flint tool industry from Bilzingsleben – Steinrinne,” in *Lower Palaeolithic Small Tools in Europe the Levant*, J. M. Burdukiewicz, A. Ronen, Eds., B.A.R British Series, (BAR Publishing, 2003), pp. 49–63.

[142] R. G. Bednarik, The Lower Paleolithic engravings of Bilzingsleben, Germany. Encyclopedia 4, 695–708 (2024).

[143] V. N. Stepanchuk, O. O. Naumenko, The earliest evidence of deliberate ivory processing dates back to around 0.4 million years ago. Int. J. Osteoarchaeol. 35, e3403 (2025).

[144] V. Dobosi, “Changing environment – Unchanged culture at Vértesszőlős, Hungary,” in *Lower Palaeolithic Small Tools in Europe and the Levant*, J. M. Burdukiewicz, A. Ronen, Eds, British Archaeological Reports, International Series 1115 (Archaeopress, 2003), pp. 101–111.

[145] P. Villa, A. P. Anzidei, E. Cerilli, “Bones and bone modifications at La Polledrara, a Middle Pleistocene site in Italy,” in *The Role of Early Humans in the Accumulation of European Lower and Middle Palaeolithic Bone Assemblages*, S. Gaudzinski, E. Turner, Eds. (Monographien des Romisch-Germanischen Zentralmuseum, 1999), vol. 42, pp. 197–206.

[146] A. P. Anzidei, “Tools from elephant bones at La Polledrara di Cecanibbio and RebibbiaCasal de’ Pazzi,” *in The World Elephants: Proceedings of the First International Congress*, Rome, G. Cavarretta, P. Gioia, M. Mussi & M. R. Palombo, Eds. 16–20 October 2001 (Consiglio Nazionale delle Ricerche, Rome, 2001), pp. 415–418.

[147] A. P. Anzidei, G. M. Bulgarelli, P. Catalano, E. Cerilli, R. Gallotti, C. Lemorini, M. Salvatore, M. R. Polombo, W. Pantano, E. Santucci, Ongoing research at the late Middle Pleistocene site of la Polledrara di Cecanibbio (Central Italy), with emphasis on human-elephant relationships. Quat. Int. 255, 171–187 (2012).

[148] C. Lemorini, E. Santucci, I. Caricola, A. Nucara, S. Nunziante-Cesaro, Life around the elephant in space and time: An integrated approach to study the human-elephant Interactions at the Late Lower Paleolithic Site of La Polledrara di Cecanibbio (Rome, Italy). J. Archaeol. Method Theory 30, 1–49 (2023).

[149] E. Cerilli, I. Fiore, Natural and anthropic events at La Polledrara di Cecanibbio (Italy, Rome): Some significant examples. Alp. Mediterr. Quat. 31, 55–58 (2018).

[150] M.-A. Julien, B. Hardy, M. C. Stahlschmidt, B. Urban, J. Serangeli, N. J. Conard, Characterizing the Lower Paleolithic bone industry from Schöningen 12 II: A multi-proxy study. J. Hum. Evol. 85, 264–286 (2015). 10.1016/j.jhevol.2015.10.006 26651609

[151] M. Domínguez-Rodrigo, “Artefactos” óseos en Torralba y Ambrona: Estudio de piezas sobre hueso post-craneal depositadas en el Museo Arqueológico Nacional. Zona Arqueológica 5, 282–287 (2005).

[152] P. Villa, F. d’Errico, Las puntas de marfil de Torralba y Ambrona. Zona Arqueológica 5, 288–304 (2005).

[153] A. Pineda, P. Saladié, The Middle Pleistocene site of Torralba (Soria, Spain): A taphonomic view of the Marquis of Cerralbo and Howell faunal collections. Archaeol. Anthropol. Sci. 11, 2539–2556 (2019).

[154] G. Wei, C. He, Y. Hu, K. Yu, S. Chen, L. Pang, Y. Wu, W. Huang, W. Yuan, First discovery of a bone handaxe in China. Quat. Int. 434, 121–128 (2017).

