# Supplementary material for: The earliest elephant-bone tool from Europe: An unexpected raw material for precision knapping of Acheulean handaxes
Source: Sci Adv. 2026 Jan 21;12(4):eady1390. doi: 10.1126/sciadv.ady1390 (PMC12822657; doi:10.1126/sciadv.ady1390)
Supplement: Supplementary file 1 — Figs. S1 to S7 Tables S1 and S2 References [file sciadv.ady1390_sm.pdf]

Science Advances
AAAS

Supplementary Materials for

The earliest elephant-bone tool from Europe: An unexpected raw material for precision knapping of Acheulean handaxes

Simon A. Parfitt and Silvia M. Bello

Corresponding author: Simon A. Parfitt, s.parfitt@ucl.ac.uk

Sci. Adv. **12**, eady1390 (2026)
DOI: 10.1126/sciadv.ady1390

This PDF file includes:

Figs. S1 to S7
Tables S1 and S2
References

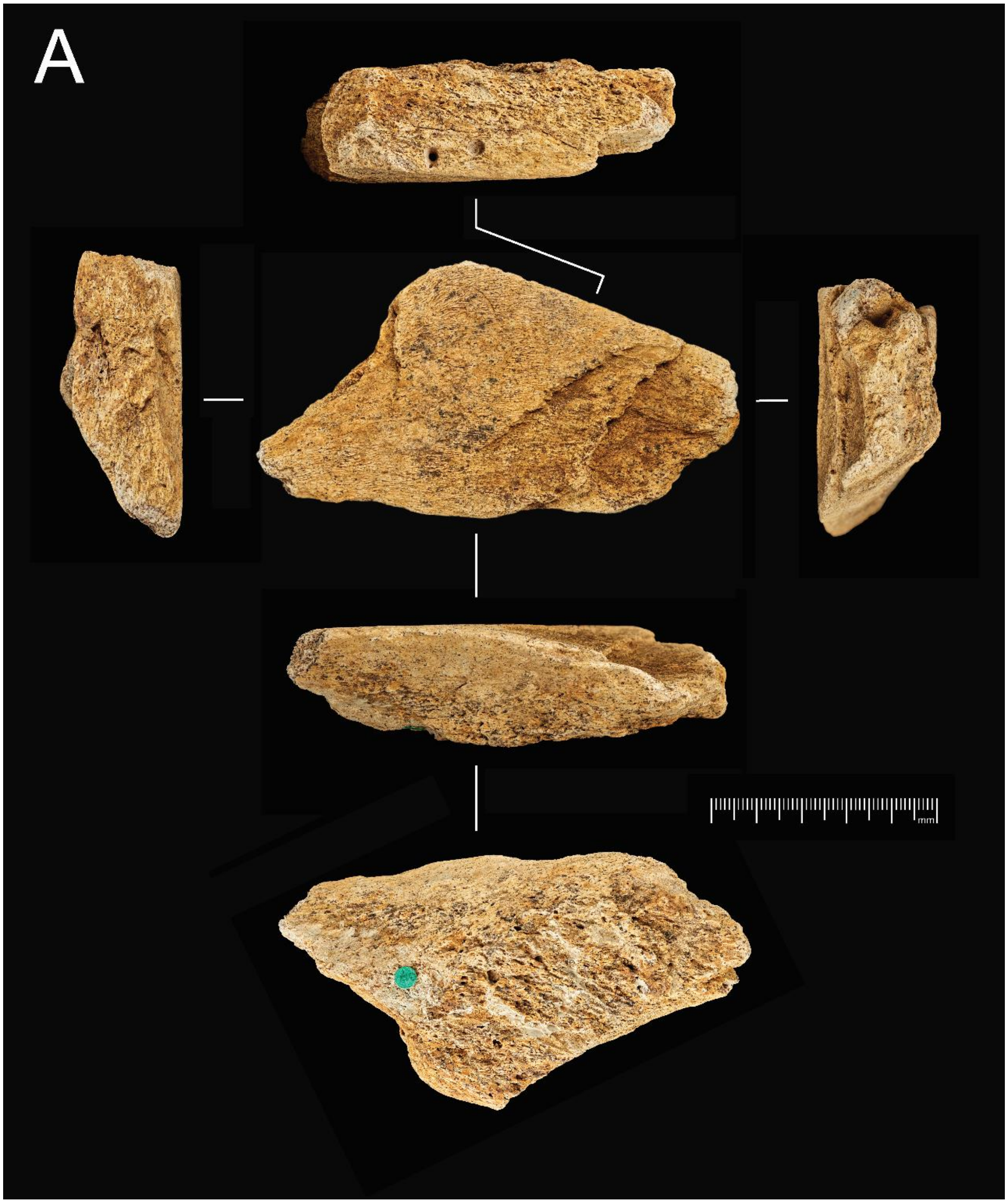
A
mm

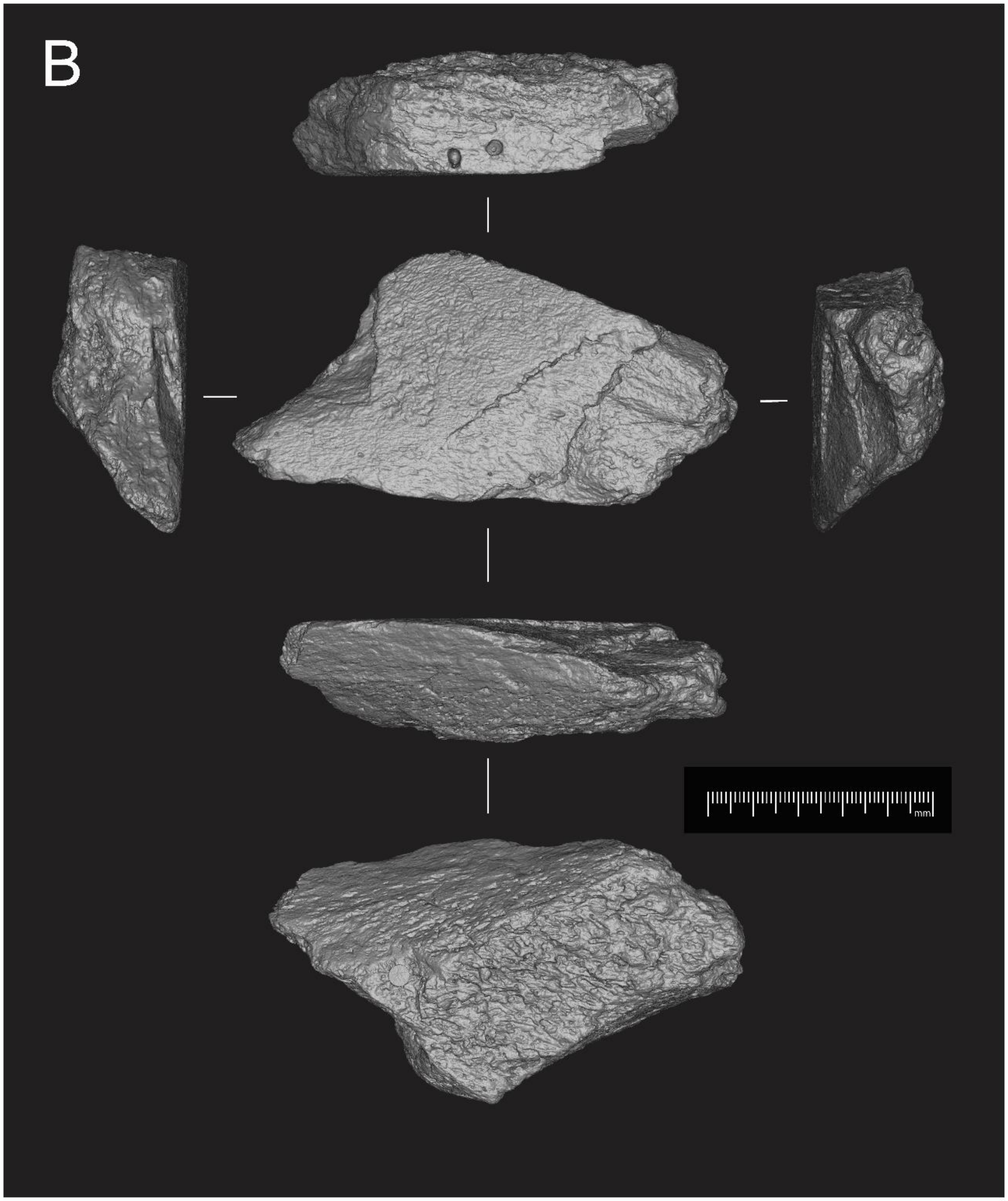

Fig. S1. Photographs (A) and 3D surface model images – orthogonal views (B) and of the Boxgrove elephant bone knapping tool (NHMUK PV UNREG. 4339). The two holes visible in the top view were made for Uranium-series dating. Copyright of the Trustees of the Natural History Museum, London.

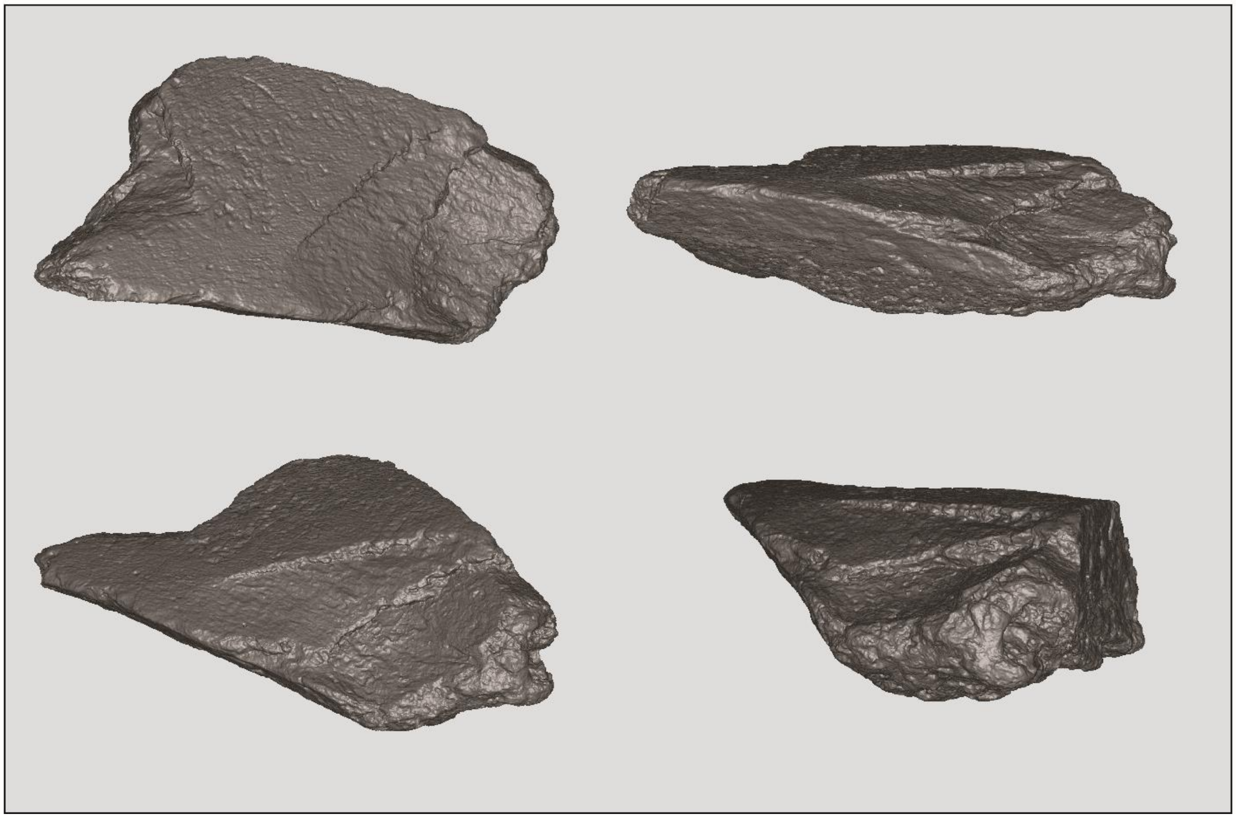

Fig. S2. 3D surface model images of flaking. Copyright of the Trustees of the Natural History Museum, London.

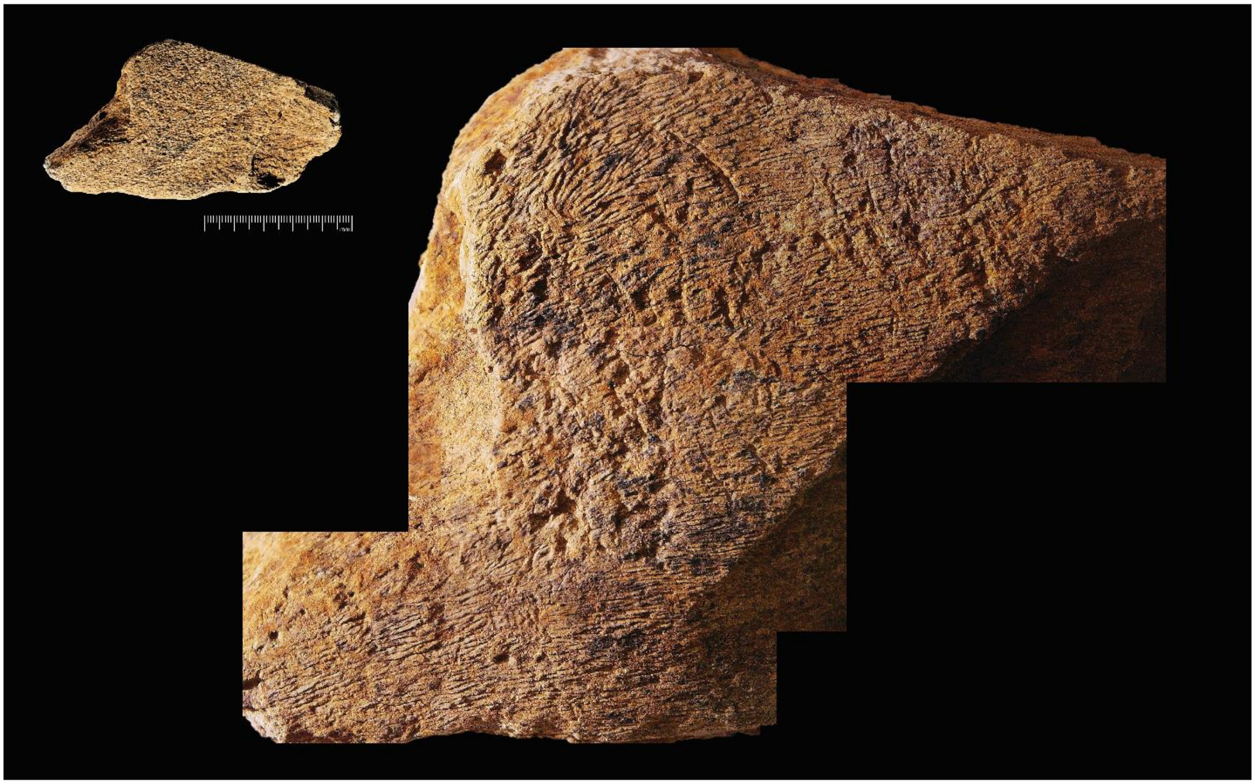

Figure S3. Photographs showing general features of the knapping marks across the three knapping areas. Copyright of the Trustees of the Natural History Museum, London.

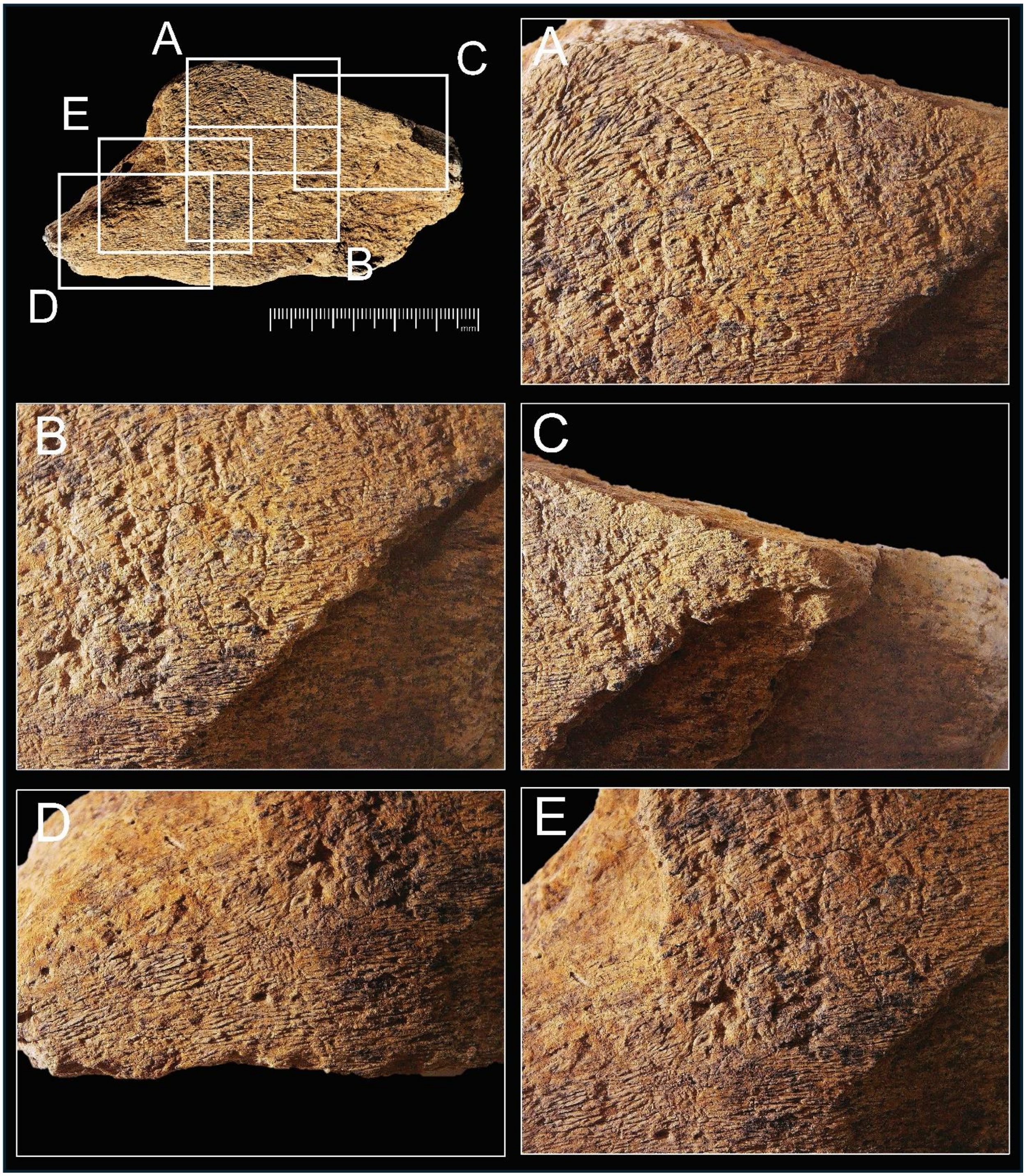

SM Figure 4. Low-magnification images of surface features. Copyright of the Trustees of the Natural History Museum, London.

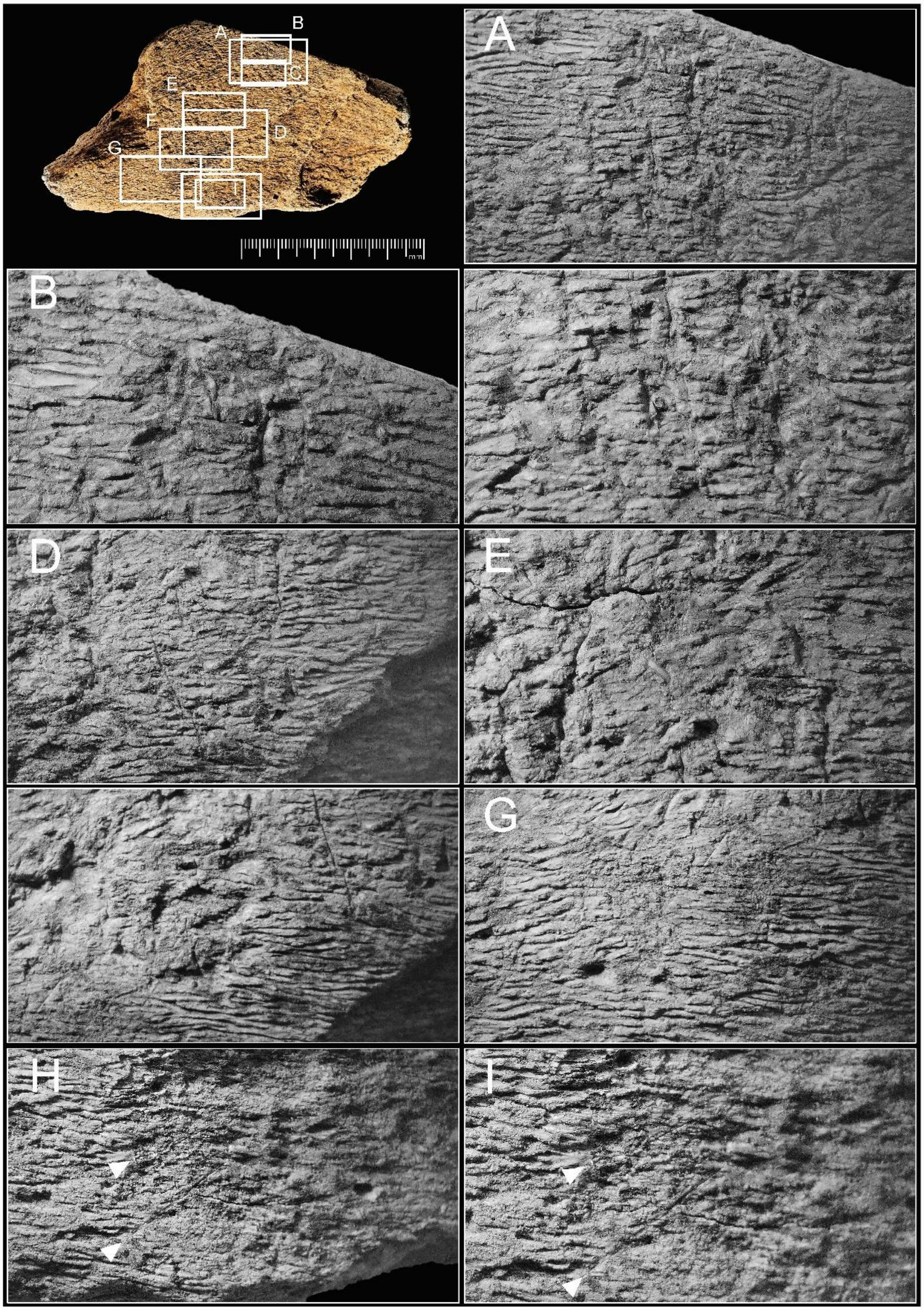

SM Figure 5. High-magnification images of surface features (A-I). White arrows (H-I) point to incisions on Flake Scar 1. Copyright of the Trustees of the Natural History Museum, London.

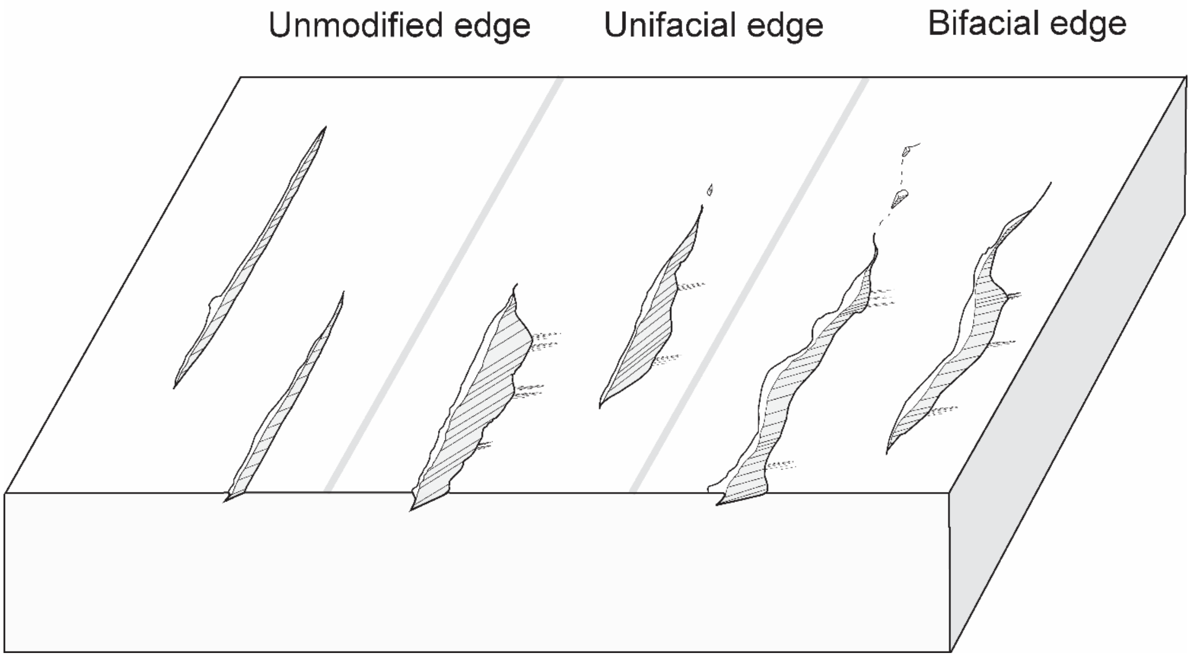

SM Figure 6. Schematic diagram showing bone modifications caused by impact against unmodified, unifacially and bifacially worked lithic edges.

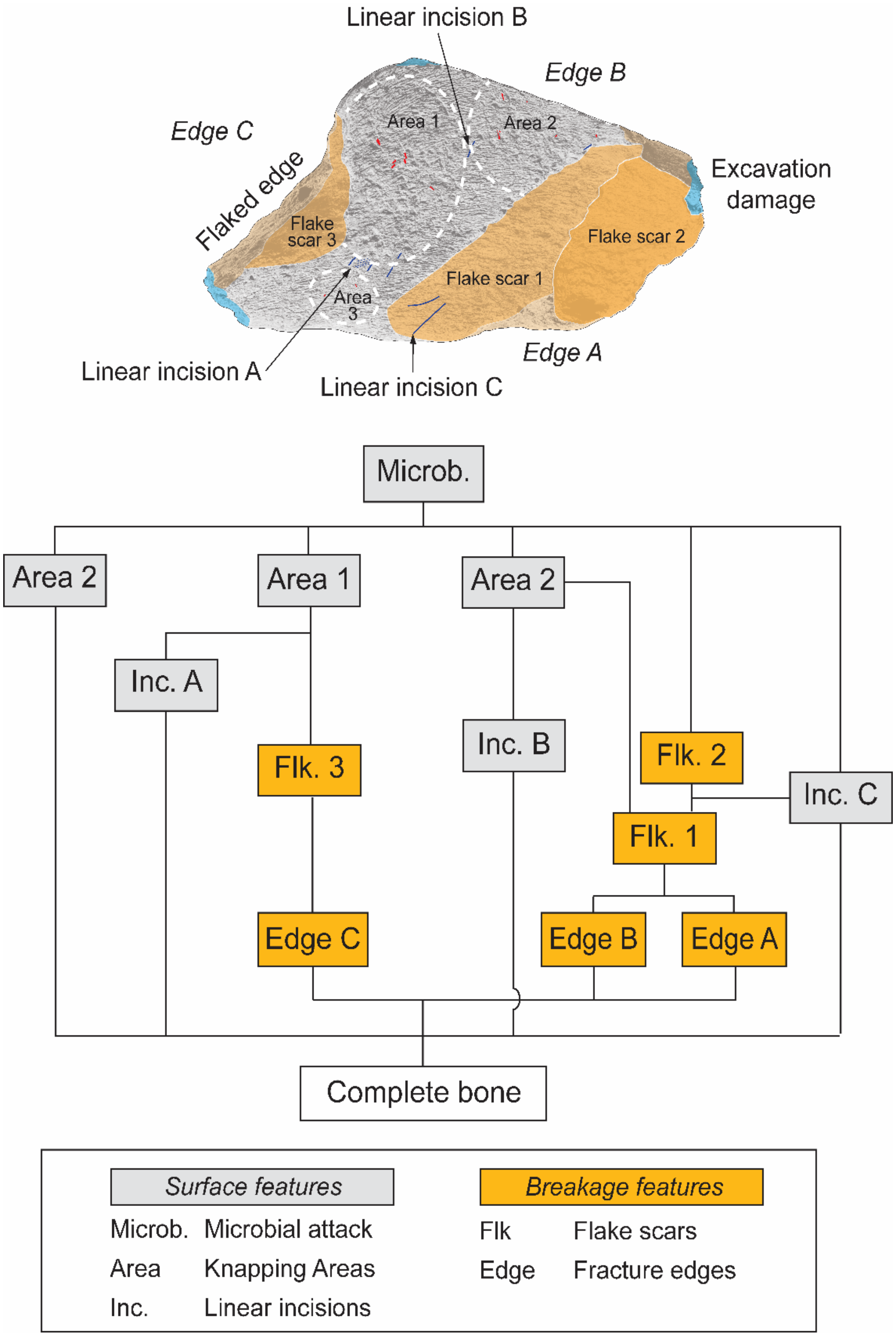

SM Figure 7. Illustration of the stages in the modification of the Boxgrove elephant bone tool using a Harris Matrix (*129*). Copyright of the Trustees of the Natural History Museum, London.

Table S1. Lower Palaeolithic sites with modified elephant bone and ivory interpreted as tools. See Figure 1 for locations.

Site	Age	Material and type	Reference
Early Pleistocene			
Olduvai Gorge Bed II (Tanzania)	1.80–1.33 Ma	Flaked bones, including a bifacially-worked piece resembling a handaxe	30-32
Olduvai Gorge Bed II (Tanzania)	~ 1.5 Ma	Bifacially-flaked cortical bone	36
Olduvai Gorge Bed IV (Tanzania)	0.8–0.5 Ma	Flaked cortical bone, including pieces resembling handaxes	35, 45
early Middle Pleistocene			
Boxgrove (UK)	~ 480 kya	Knapping tool	This paper
late Middle Pleistocene			
Marathousa I (Greece)	478–424 kya	Flaked bone with percussion marks	49
Malagrotta (Lazio, Italy)	451–378 kya	Bone handaxe	130, 131
Revadim Quarry (Israel)	?500–300 kya	2 bone handaxes, bifacially-worked bone resembling biface, wedge	132, 133
Isoletta (Ceprano Basin, Lazio, Italy)	430–310 kya	Use-wear on cortical flakes	37, 134
Colle Avarone (Ceprano Basin, Lazio, Italy)	430–410 kya	Modified elephant rib interpreted as a 'dagger'); use-wear on cortical flakes	37, 134, 135
Rome, Via Aurelia Km 19 (Lazio, Italy)	412–325 kya	17 flaked diaphyseal fragments	135
Selvotta (Ceprano Basin, Lazio, Italy)	410–350 kya	Use-wear on cortical flakes	37, 134
Pofi, Cava Pompi (Tuscany, Italy)	~ 407 kya	5 possible bone tools (1 flaked bone, 3 fragments of diaphysis with 'worn' tips, and 1 non-specific tool)	135
Fontana Rannucchio (Lazio, Italy)	~ 407 kya	Bone bifaces, flaked cortical bone fragments resembling handaxes, retouched bones on elephant bone fragments, indented pelvis interpreted as an anvil	135-137
Castel di Guido (Lazio, Italy)	~ 400 kya	Numerous Acheulean handaxes, smoothers, unifaces and pointed tools, wedges	130, 138
Lademagne (Lazio, Italy)	404–388 kya	Modified diaphysis fragments and rib	40, 130, 137
Casal Lumbroso	~ 404 kya	Intentionally fractured elephant bone fragments with localised use wear traces	139
Bilzingsleben (Germany)	? 424–374 kya	Bone bifaces and other bone and ivory artefacts in a variety of flaked, polished and 'engraved' forms	140-142
Medzhibozh A (Ukraine)	424–374 kya	Minute chipped ivory objects	143
Pontecorvo (Lazio, Italy)	Not dated (Acheulean industry)	Scraper from elephant bone diaphysis	130, 135
Vérteszöllös (Hungary)	late Middle Pleistocene	Bone biface and other bone tools	144
La Polledrara di Cecanibbio (Italy)	~ 324 kya	Retouched tools made of elephant diaphyses	145-149
Schöningen (Germany)	~ 300 kya	Fractured tusk fragment with smoothed and polished tip (?tool)	150
Casal de' Pazzi (Rebibbia, Lazio, Italy)	243–191 kya	Cortical bone with unifacial flaking	146
Torralba and Ambrona (Spain)	> 200 kya	Unspecified number of purported bone tools including bifacially flaked pieces	151-153
Baishiyi (China)	~ 170 kya	'Proto-handaxe'	154

Table S2. Elephant remains from the Boxgrove Waterhole Site (Q1/B), 1993-96 excavations. The cheek tooth fragment is a tabular ‘plate’ of elephantid enamel. The tusk fragments (NHMUK PV M 119811–119824) consist of small, shattered pieces of ivory, separated along concentric ‘lines of Owen’. BR = broken during excavation. The tusk tip (NHMUK PV M 119836) was found at a lower level than the other pieces and is probably from a different tusk.

NHMUK PV	Site number Q1/B	Element	Length (mm)	Width (mm)	Unit	Spit	Sq. E	Sq. N	m O.D.
M 119836	F 3344	Tusk (tip)	15	18	4	1	205	110	40.55
UNREG. 4339	F A317	Indeterminate cortical bone frag. (knapping tool)	110	60	4b	-	-	-	-
M 119805	F 269	Vertebral epiphysis frag. (unfused)	30	24	8a	9	202	101	40.72
M 119806	F 370	Vertebral epiphysis frag. (unfused)	42	39	8a	10	201	103	40.59
M 119807	F 4272	Vertebral epiphysis frag. (unfused)	27	19	8ac	7	201	97	40.03
M 119808	F 4273	Vertebral epiphysis frag. (unfused)	119	76	8ac	7	201	95	40.08
M 119809	F 4277	Vertebral epiphysis frag. (unfused)	18	11	8ac	7	201	95	39.99
M 119810	F 4280	Vertebral epiphysis frag. (unfused)	23	22	8ac	7	201	94	39.98
M 119709	F 4483	Cheek tooth frag.	29	10	8ac	2	206	94	40.11
M 119811	F 4297	Tusk frag.	39	17	8ac	1	205	95	40.10
M 119812	F 3954	Tusk frag.	16	10	8ac	1	202	97	40.25
M 119813	F 4300	Tusk frag.	33	15	8ac	1	205	96	40.15
M 119814	F 4303	Tusk frag.	31	22	4d1	1	205	98	40.20
M 119815	F 4312	Tusk frag.	69	16	8ac	2	203	96	40.26
M 119816	F 4467	Tusk frag.	BR	BR	8ac	2	205	95	40.11
M 119817	F 4476	Tusk frag.	24	6	8ac	2	206	94	40.12
M 119818	F 4544	Tusk frag.	40	12	8ac	3	205	94	40.04
M 119819	F 4545	Tusk frag.	29	13	8ac	3	205	94	40.03
M 119820	F 4548	Tusk frag.	BR	BR	8ac	3	205	94	40.06
M 119821	F 4565	Tusk frag.	22	9	8ac	5	203	94	40.06
M 119822	F 4799	Tusk frag.	17	14	4	4	205	94	39.94
M 119823	F 5043	Tusk frag.	25	16	4	7	219	94	39.81
M 119824	F 5065	Tusk frag.	43	23	4	5	206	94	39.93

REFERENCES

1. A. M. Lister, A. J. Stuart, The West Runton mammoth (*Mammuthus trogontherii*) and its evolutionary significance. *Quat. Internat.* **228**, 80–209 (2010).

2. E. E. Erkek, A. M. Lister, The skeleton of a straight-tusked elephant, *Palaeoloxodon antiquus* (Falconer and Cautley, 1847) from Selsey, England, and growth and variation in *Palaeoloxodon* of the European Pleistocene. *J. Quat. Sci.* **36**, 211–223 (2021).

3. A. J. Stuart, *Vanished Giants. The Lost World of the Ice Age* (The University of Chicago Press, 2021).

4. T. Hauffe, J. L. Cantalapiedra, D. Silvestro, Trait-mediated speciation and human-driven extinctions in proboscideans revealed by unsupervised Bayesian neural networks. *Sci. Adv.* **10**, eadl2643 (2024).

5. G. M. Bhat, N. Ashton, S. Parfitt, A. Jukar, M. R. Dickinson, B. Thusu, J. Craig, Human exploitation of a straight-tusked elephant (*Palaeoloxodon*) in Middle Pleistocene deposits at Pampore, Kashmir, India. *Quat. Sci. Rev.* **342**, 108894 (2024).

6. S. Gaudzinski-Windheuser, L. Kindler, K. MacDonald, W. Roebroeks, Hunting and processing of straight-tusked elephants 125.000 years ago: Implications for Neanderthal behavior. *Sci. Adv.* **9**, eadd8186 (2023).

7. S. Gaudzinski-Windheuser, L. Kindler, W. Roebroeks, Widespread evidence for elephant exploitation by Last Interglacial Neanderthals on the North European plain. *Proc. Natl. Acad. Sci. U.S.A.* **120**, e2309427120 (2023).

8. S. Gaudzinski, E. Turner, A. P. Anzidei, E. Alvarez-Fernández, J. Arroyo-Cabrales, J. Cinq-Mars, V. T. Dobosi, A. Hannus, E. Johnson, S. C. Munzel, A. Scheer, P. Villa, The use of proboscidean remains in every-day Palaeolithic life. *Quat. Int.* **126-128**, 179–194 (2005).

9. G. Boschian, D. Saccà, In the elephant, everything is good: Carcass use and re-use at Castel di Guido (Italy). *Quat. Int.* **361**, 288–296 (2015).

10. L. Buck, C. Stringer, Having the stomach for it: A contribution to Neanderthal diets? *Quat. Sci. Rev.* **96**, 161–167 (2014).

11. G. E. Konidaris, R. Barkai, V. Tourloukis, K. Harvati, Eds., *Human-Elephant Interactions: From Past To Present* (Tübingen Univ. Press, 2021).

12. G. Boschian, D. Caramella, D. Saccà, R. Barkai, Are there marrow cavities in Pleistocene elephant limb bones, and was marrow available to early humans? New CT scan results from the site of Castel di Guido (Italy). *Quat. Sci. Rev.* **215**, 86–97 (2019).

13. A. Lister, P. G. Bahn, *Mammoths: Giants of the Ice Age* (University of California Press, 2007).

14. N. J. Conard, M. Malina, S. C. Münzel, New flutes document the earliest musical tradition in southwestern Germany. *Nature* **460**, 737–740 (2009).

15. P. Valde-Nowak, A. Nadachowski, M. Wolsan, Upper Palaeolithic boomerang made of a mammoth tusk in south Poland. *Nature* **329**, 436–438 (1987).

16. N. J. Conard, Palaeolithic ivory sculptures from southwestern Germany and the origins of figurative art. *Nature* **426**, 830–832 (2003).

17. C.-J. Kind, N. Ebinger-Rist, S. Wolf, T. Beutelspacher, K. Wehrberger, The smile of the Lion Man. Recent excavations in Stadel Cave (Baden-Württemberg, southwestern Germany) and the restoration of the famous Upper Palaeolithic figurine. *Quartär* **61**, 129–145 (2014).

18. M. Oliva, "The Brno II Upper Palaeolithic burial," in *Hunters of the Golden Age*, W. Roebroeks, M. Mussi, J. Svoboda, K. Fennema, Eds. (Faculty of Archaeology, 2000), pp. 143–159.

19. M. Sablin, N. Reynolds, K. Iltsevich, M. Germonpréc, The Epigravettian site of Yudinovo, Russia: Mammoth bone structures as ritualised middens. *Environ. Archaeol.* **30**, 1–21 (2023).

20. O. Soffer, *The Upper Paleolithic of the Central Russian Plain* (Academic Press, 1985).

21. R. G. Klein, *Ice-Age Hunters of the Ukraine* (The University of Chicago Press, 1973).

22. P. Callow, J. M. Cornford, Eds., *La Cotte de St Brelade 1961–1978. Excavations by C.B.M. McBurney* (Geobooks, 1986).

23. K. Scott, Two hunting episodes of Middle Palaeolithic age at La Cotte de Saint-Brelade, (Channel Islands), Jersey. *World Archaeol.* **12**, 137–152 (1980).

24. K. Scott, "The bone assemblages from layers 3 and 6," in *La Cotte de St Brelade 1961–1978. Excavations by C.B.M. McBurney*, P. Callow, J. M. Cornford, Eds. (Geobooks, 1986), pp. 159–185.

25. B. Scott, M. Bates, R. Bates, C. Conneller, M. Pope, A. Shaw, G. Smith, A new view from La Cotte de St Brelade, Jersey. *Antiquity* **88**, 13–29 (2014).

26. B. Scott, A. Shaw, K. Scott, M. Pope, *Repeopling La Manche: New Perspectives on Neanderthal Lifeways from La Cotte de St Brelade*, Prehistoric Society Research Paper 10 (The Prehistoric Society, 2023).

27. A. Shaw, B. Scott, M. Pope, "The early Middle Palaoelithic 'bone heaps' from La Cotte de St Brelade reconsidered," in *Repeopling La Manche: New Perspectives on Neanderthal Lifeways from La Cotte de St Brelade*, B. Scott, A. Shaw, K. Scott, M. Pope, Eds., Prehistoric Society Research Paper 10 (The Prehistoric Society, 2022), pp. 43–64.

28. C. B. Stringer, *Homo Britannicus: The Incredible Story of Human Life in Britain*. (Penguin Books Ltd., 2007), vol. 27, pp. 39–40.

29. F. Wenban-Smith, "The essential elephant: Northwest European Hominin adaptations through the Middle-Late Pleistocene and Neanderthal extinction," in *Human-Elephant Interactions from Past to Present*, G. E. Konidaris, R. Barkai, V. Tourloukis, K. Harvati, Eds. (Tübingen Univ. Press, Tübingen, 2021), pp. 145–176.

30. M. D. Leakey, *Olduvai Gorge: Excavations in Bed I and II, 1960–1963* (Cambridge Univ. Press, 1971).

31. P. Shipman, "Altered bones from Olduvai Gorge, Tanzania: Techniques, problems and implications for their recognition," in *Bone Modification*, R. Bonnichsen, M. H. Sorg, Eds. (University of Maine Centre for the Study of the First Americans, Thompson-Shore Inc., 1989), pp. 317–334.

32. L. R. Backwell, F. d'Errico, The first use of bone tools: A reappraisal of the evidence from Olduvai Gorge, Tanzania. *Palaeontol. Afr.* **40**, 95–158 (2004).

33. L. Backwell, F. d'Errico, "The origin of bone tool technology and the identification of early hominid cultural traditions," in *From Tools to Symbols: From Early Hominids to Modern Humans*, F. d'Errico, L. Backwell, Eds. (Wits Univ. Press, 2005), pp. 238–275.

34. L. R. Backwell, F. d'Errico, "Palaeolithic bone tools," in *Encyclopedia of Global Archaeology*, C. Smith, Ed. (Springer, 2014), pp. 950–962.

35. M. Pante, I. de la Torre, F. d'Errico, J. Njau, R. Blumenschine, Bone tools from Beds II-IV, Olduvai Gorge, Tanzania, and implications for the origins and evolution of bone technology. *J. Hum. Evol.* **148**, 102885 (2020).

36. I. de la Torre, L. Doyon, A. Benito-Calvo, R. Mora, I. Mwakyoma, J. K. Njau, R. F. Peters, A. Theodoropoulou, F. d'Errico, Systematic bone tool production at 1.5 million years ago. *Nature* **640**, 130–134 (2025).

37. F. Marinelli, M.-H. Moncel, C. Lemorini, The use of bones as tools in Late Lower Paleolithic of Central Italy. *Sci. Rep.* **14**, 11666 (2024).

38. K. Zutovski, R. Barkai, The use of elephant bones for making Acheulian handaxes: A fresh look at old bones. *Quat. Int.* **406**, 227–238 (2016).

39. R. Barkai, The elephant in the handaxe: Lower Palaeolithic ontologies and representations. *Camb. Archaeol. J.* **31**, 349–361 (2021).

40. I. Biddittu, P. Celletti, "Plio-Pleistocene Proboscidea and Lower Palaeolithic bone industry of southern Latium (Italy)," in *The World of Elephants* (International Congress, 2001), pp. 91–96.

41. A. G. Costa, “A geometric morphometric assessment of plan shape in bone and stone Acheulean bifaces from the Middle Pleistocene site of Castel di Guido, Latium, Italy,” in *New Perspectives on Old Stones*, S. Lycett, P. Chauhan, Eds. (Springer, 2010), pp. 23–41.

42. S. A. Semenov, *Prehistoric Technology: An Experimental Study of the Oldest Tools and Artefacts from Traces of Manufacture and Wear*, M. W. Thompson, Transl. (Cory, Adams & Mackay, London, 1964).

43. G. Haynes, *Mammoths, Mastodonts, and Elephants: Biology, Behavior, and the Fossil Record* (Cambridge Univ. Press, 1991).

44. J. M. Hutson, A. García-Moreno, E. S. Noack, E. Turner, A. Villaluenga, S. Gaudzinski-Windheuser, “The origins of bone tool technologies: Conclusions and future directions,” in *The Origins of Bone Tool Technologies*, J. M. Hutson, A. García-Moreno, E. S. Noack, E. Turner, A. Villaluenga, S. Gaudzinski-Windheuser, Eds. (RGZM, 2018), pp. 317–326.

45. M. D. Leakey, D. A. Roe, *Olduvai Gorge 5: Excavations in Beds III, IV and the Masek Beds, 1968–1971* (Cambridge Univ. Press, 1994).

46. J. K. Njau, R. J. Blumenschine, A diagnosis of crocodile feeding traces on larger mammal bone, with fossil examples from the Plio-Pleistocene Olduvai Basin, Tanzania. *J. Hum. Evol.* **50**, 142–162 (2006).

47. J. K. Njau, H. G. Gilbert, “Standardizing terms for crocodile-induced bite marks on bone surfaces in light of the frequent bone modification equifinality found to result from crocodile feeding behavior, stone tool modification, and trampling,” in *FOROST Occasional Publication* (2016), vol. 3, pp. 1–13.

48. Y. Sahle, S. El Zaatari, T. D. White, Hominid butchers and biting crocodiles in the African Plio-Pleistocene. *Proc. Natl. Acad. Sci. U.S.A.* **114**, 13164–13169 (2017).

49. V. Tourloukis, N. Thompson, E. Panagopoulou, D. Giusti, G. E. Konidaris, P. Karkanas, K. Harvatia, Lithic artifacts and bone tools from the Lower Palaeolithic site Marathousa 1, Megalopolis, Greece: Preliminary results. *Quat. Int.* **497**, 47–64 (2018).

50. S. M. Bello, S. A. Parfitt, Taphonomic approaches to distinguish chewing damage from knapping marks in Palaeolithic faunal assemblages. *J. Archaeol. Sci. Rep.* **51**, 104183 (2023).

51. A. Woodcock, *The Lower and Middle Palaeolithic of Sussex*, B.A.R British Series 94 (BAR Publishing, 1981).

52. M. B. Roberts, S. A. Parfitt, M. I. Pope, F. F. Wenban-Smith, R. I. Macphail, A. Locker, J. R. Stewart, Boxgrove, West Sussex: Rescue excavations of a Lower Palaeolithic landsurface (Boxgrove Project B, 1989–1991). *Proc. Prehist. Soc.* **63**, 303–358 (1997).

53. M. B. Roberts, S. A. Parfitt, *Boxgrove. A Middle Pleistocene Hominid Site at Eartham Quarry, Boxgrove, West Sussex*, English Heritage Archaeological Report 17 (English Heritage, 1999).

54. M. Pope, S. Parfitt, M. Roberts, *The Horse Butchery Site: A High-Resolution Record of Lower Palaeolithic Hominin Behaviour at Boxgrove, UK* (SpoilHeap Publications, 2020).

55. R. C. Preece, S. A. Parfitt, Environmental heterogeneity of the Lower Palaeolithic land surface on the Goodwood-Slindon Raised Beach: Comparisons of the records from Boxgrove and Valdoe, Sussex, UK. *J. Quat. Sci.* **37**, 572–592 (2022).

56. S. A. Parfitt, R. C. Preece, New palaeontological evidence suggests an early Middle Pleistocene age for the lower levels of Sun Hole Cave, Cheddar, Somerset, UK. *Proc. Geol. Assoc.* **133**, 162–175 (2022).

57. C. B. Stringer, E. Trinkaus, M. B. Roberts, S. A. Parfitt, R. I. Macphail, The Middle Pleistocene human tibia from Boxgrove. *J. Hum. Evol.* **34**, 509–547 (1998).

58. S. Hillson, S. A. Parfitt, S. M. Bello, M. B. Roberts, C. B. Stringer, Two hominin incisor teeth from the Middle Pleistocene site of Boxgrove, Sussex, England. *J. Hum. Evol.* **59**, 493–503 (2010).

59. A. L. Lockey, L. Rodríguez, L. Martín-Francés, J. L. Arsuaga, J. M. Bermúdez de Castro, L. Crété, M. Martinón-Torres, S. Parfitt, M. Pope, C. Stringer, Comparing the Boxgrove and

Atapuerca (Sima de los Huesos) human fossils: Do they represent distinct paleodemes? *J. Hum.Evol.* **172**, 10325 (2022).

60. M. W. Pitts, M. Roberts, *Fairweather Eden: Life in Britain Half a Million Years Ago as Revealed by the Excavations at Boxgrove* (Century, 1997).

61. S. A. Parfitt, S. M. Bello, "The manufacture and use of bone tools," in *The Horse Butchery Site: A High-resolution Record of Lower Palaeolithic Hominin Behaviour at Boxgrove, UK*, M. Pope, S. Parfitt, M. Roberts, Eds. (SpoilHeap Publications, 2020), pp. 105–121.

62. S. A. Parfitt, S. M. Bello, Bone tools, carnivore chewing and heavy percussion: Assessing conflicting interpretations of Lower and Upper Palaeolithic bone assemblages. *R. Soc. Open Sci.* **11**, 11231163 (2024).

63. S. A. Parfitt, M. D. Lewis, S. M. Bello, Taphonomic and technological analyses of Lower Palaeolithic bone tools from Clacton-on-Sea, UK. *Sci. Rep.* **12**, 20222 (2022).

64. L. Sánchez-Romero, A. Benito-Calvo, D. De Loecker, M. Pope, Spatial analysis and site formation processes associated with the Middle Pleistocene hominid teeth from Q1/B waterhole, Boxgrove (West Sussex, UK). *Archaeol. Anthropol. Sci.* **15**, 98 (2023).

65. M. Pope, "*The Significance of Biface-rich Assemblages: An Examination of Behavioural Control on Lithic Assemblage Formation in the Lower Palaeolithic*", thesis, University of Southampton, UK (2001).

66. P. García-Medrano, A. Ollé, N. Ashton, M. B. Roberts, The mental template in handaxe manufacture: New insights into Acheulean lithic technological behavior at Boxgrove, Sussex, UK. *J. Archaeol. Method Theory* **26**, 396–422 (2019).

67. J. C. Mitchell, "A use-wear analysis of selected British Lower Palaeolithic handaxes with special reference to the site of Boxgrove (West Sussex): A study incorporating optical microscopy, computer aided image analysis and experimental archaeology," thesis, University of Oxford, UK (1998).

68. R. Iovita, S. P. McPherron, The handaxe reloaded: A morphometric reassessment of Acheulian and Middle Paleolithic handaxes. *J. Hum. Evol.* **61**, 61–74 (2011).

69. C. Shipton, F. Foulds, A. Rawlinson, M. Leroyer, N. Ashton, M. White, Turning-the-edge, tranchet, and social signalling at Boxgrove. *Cam. Archaeol. J.* **2025**, 1–18 (2025).

70. C. Shipton, C. Clarkson, R. Cobden, Were Acheulean bifaces deliberately made symmetrical? Archaeological and experimental evidence. *Camb. Archaeol. J.* **29**, 65–79 (2019).

71. F. F. Wenban-Smith, The use of canonical variates for determination of biface manufacturing technology at Boxgrove Lower Palaeolithic site and the behavioural implications of this technology. *J. Archaeol. Sci.* **16**, 17–26 (1989).

72. D. Stout, J. Apel, J. Commander, M. Roberts, Late Acheulean technology and cognition at Boxgrove, UK. *J. Archaeol. Sci.* **41**, 576–590 (2014).

73. M. B. Roberts, S. A. Parfitt, "The bone hammers from the early Middle Pleistocene site at Boxgrove, West Sussex, UK: Their identification, process of manufacturing, and use," in *Retouching the Palaeolithic: Becoming Human and the Origins of Bone Tool Technology*, J. Hutson, A. García-Moreno, A. Villaluenga, Eds. (MONREPOS Research Centre and Museum for Human Behavioural Evolution – RGZM, 2015), p. 48.

74. A. V. Neal, "The analysis and investigation of partially fossilized bone material from the Lower Palaeolithic site at Amey's Eartham Pit, Boxgrove, West Sussex", thesis, University of London, UK (1987).

75. M. M. E. Jans, "Microscopic destruction of bone," in *Manual of Forensic Taphonomy*, J. T. Pokines, E. N. L'Abbé, S. A. Symes, Eds. (CRC Press, 2021), pp. 23–40.

76. M. M. E. Jans, C. M. Nielsen-Marsh, C. I. Smith, M. J. Collins, H. Kars, Characterisation of microbial attack on archaeological bone. *J. Archaeol. Sci.* **31**, 87–95 (2004).

77. C. J. Hackett, Microscopical focal destruction (tunnels) in exhumed human bones. *Med. Sci. Law* **21**, 243–265 (1981).

78. V. Marchiafava, E. Bonucci, A. Ascenzi, Fungal osteoclasia: A model of dead bone resorption. *Calcif. Tissue Int.* **14**, 195–210 (1974).

79. Y. Fernández-Jalvo, P. Andrews, D. Pesquero, C. Smith, D. Marín-Monfort, B. Sánchez, E.-M. Geigl, A. Alonso, Early bone diagenesis in temperate environments. Part I: Surface features and histology. *Palaeogeogr. Palaeoclimatol. Palaeoecol.* **288**, 62–81 (2010).

80. G. Turner-Walker, Light at the end of the tunnels? The origins of microbial bioerosion in mineralised collagen. *Palaeogeogr. Palaeoclimatol. Palaeoecol.* **529**, 24–38 (2019).

81. A. M. Lister, The stratigraphical significance of deer species in the Cromer Forest-bed Formation. *J. Quat. Sci.* **8**, 95–108 (1993).

82. A. M. Lister, "Ecological Interactions of Elephantids in Pleistocene Eurasia: *Palaeoloxodon* and *Mammuthus*," in *Human Paleoecology in the Levantine Corridor*, N. Goren-Inbar, J. D. Speth, Eds. (Oxbow Books, 2017), pp. 53–60.

83. A. M. Lister, Dating the arrival of straight-tusked elephant (*Palaeoloxodon* spp.) in Eurasia. *Bulletin Du Musée d'anthropologie Préhistorique De Monaco Supplément* **6**, 123–128 (2016).

84. R. Potts, P. Shipman, Cutmarks made by stone tools on bones from Olduvai Gorge, Tanzania. *Nature* **291**, 577–580 (1981).

85. Y. Fernández-Jalvo, P. Andrews, *Atlas of Taphonomic Identifications. 1001+ Images of Fossil and Recent Mammal Bone Modification* (Springer, 2016).

86. T. van Kolfschoten, S. A. Parfitt, J. Serangeli, S. M. Bello, Lower Paleolithic bone tools from the "Spear Horizon" at Schöningen (Germany). *J. Hum. Evol.* **89**, 226–263 (2015).

87. J. M. Hutson, A. García-Moreno, E. S. Noack, E. Turner, A. Villaluenga, S. Gaudzinski-Windheuser, Eds., *The Origins of Bone Tool Technologies* (RGZM, 2018).

88. M. É. Dupont, *Les Temps Antéhistoriques en Belgique: L'Homme Pendant les Âges de la Pierre dans les Environs de Dinant-sur-Meuse* (Muquardt, 1871).

89. F. Daleau, “Sur des lésions que présentent certains os de la période paléolithique”, in *Association Française pour l’Avancement des Sciences, Compte Rendu de la 12e Session, Rouen 1883* (Chaix, 1884), pp. 600–602.

90. M. Patou-Mathis, Ed., *Retouchoirs, Compresseurs, Percuteurs. Os à Impressions et à Éraillures. Fiches Typologiques de l’Industrie Osseuse Préhistorique, Cahier X* (Éditions Société Préhistorique Française, 2002).

91. S. M. Bello, G. Delbarre, I. De Groote, S. A. Parfitt, A newly discovered antler flint-knapping hammer and the question of their rarity in the Palaeolithic archaeological record: Reality or bias? *Quat. Int.* **403**, 107–117 (2016).

92. J. B. Mallye, C. Thiébaut, V. Mourre, S. Costamagno, É. Claud, P. Weisbecker, The Mousterian bone retouchers of Noisetier Cave: Experimentation and identification of marks. *J. Archaeol. Sci.* **39**, 1131–1142 (2012).

93. A. K. Behrensmeyer, The bones of Amboseli: Bone assemblages and ecological change in a modern African ecosystem. *Natl. Geogr. Res.* **9**, 402–421 (1993).

94. G. Haynes, Longitudinal studies of African elephant death and bone deposits. *J. Archaeol. Sci.* **15**, 131–157 (1988).

95. G. Haynes, K. Krasinski, P. Wojtal, A study of fractured proboscidean bones in recent and fossil assemblages. *J. Archaeol. Method Theory* **28**, 956–1025 (2021).

96. L. Douglas-Hamilton, O. Doulas-Hamilton, *Among the Elephants* (The Viking Press, 1975).

97. A. J. Stuart, N. Larkin, Taphonomy of the West Runton Mammoth. *Quat. Int.* **228**, 217–232 (2010).

98.P Biberson, E. Aguirrre, Experiences de taille d'outils préhistoriques dans des os d’élephant. *Quaternaria*, 7, 165–183 (1965).

99. S. J. Lycett, J. A. J. Gowlett, On questions surrounding the Acheulean ‘tradition’. *World Archaeol.* **40**, 295–315 (2008).

100. M. Mussi, J. Panera, S. Rubio-Jara, T. W. Davies, D. Geraads, H. Bocherens, G. Briatico, A. Le Cabec, J.-J. Hublin, A. Gidna, R. Bonnefille, L. Di Bianco, E. Méndez-Quintas, Early *Homo erectus* lived at high altitudes and produced both Oldowan and Acheulean tools. *Science* **382**, 713–718 (2023).

101. R. G. Klein, The earlier stone age of Southern Africa. *S. Afr. Archaeol. Bull.* **55**, 107–122 (2000).

102. R. Klein, "Hominin dispersals in the Old World," in *The Human Past. World Prehistory & the Development of Human Societies*, C. Scarre, Ed. (Thames & Hudson Ltd., ed. 3, 2013), pp. 84–123.

103. O. Bar-Yosef, M. Belmaker, Early and Middle Pleistocene Faunal and hominins dispersals through Southwestern Asia. *Quat. Sci. Rev.* **30**, 1318–1337 (2011).

104. I. de la Torre, The origins of the Acheulean: Past and present perspectives on a major transition in human evolution. *Phil. Trans. R. Soc.* **371**, 20150245 (2016).

105. G. R. Scott, L. Gibert, The oldest hand-axes in Europe. *Nature* **461**, 82–85 (2009).

106. X. Li, H. Ao, M. J. Dekkers, A. P. Roberts, P. Zhang, S. Lin, W. Huang, Y. Hou, W. Zhang, Z. An, Early Pleistocene occurrence of Acheulian technology in North China. *Quat. Sci. Rev.* **156**, 12–22 (2017).

107. H. Li, K. Kuman, C. Li, What is currently (un)known about the Chinese Acheulean, with implications for hypotheses on the earlier dispersal of hominids. *Comptes Rendus Palevol* **17**, 120–130 (2018).

108. H. Yamei, R. Potts, Y. Baoyin, G. Zhengtang, A. Deino, W. Wei, J. Clark, X. Guangmao, H. Weiwen, Mid-Pleistocene Acheulean-like stone technology of the Bose Basin, South China. *Science* **287**, 1622–1626 (2000).

109. S. Pappu, Y. Gunnell, K. Akhilesh, R. Braucher, M. Taieb, F. Demory, N. Thouveny, Early Pleistocene presence of Acheulian hominins in South India. *Science* **331**, 1596–1599 (2011).

110. J. Galway-Witham, J. Cole, C. Stringer, Aspects of human physical and behavioural evolution during the last 1 million years. *J. Quat. Sci.* **34**, 355–378 (2019).

111. A. J. M. Key, I. Jarić, D. L. Roberts, Modelling the end of the Acheulean at global and continental levels suggests widespread persistence into the Middle Palaeolithic. *Humanit. Soc. Sci. Commun.* **8**, 1–12 (2021).

112. J. Wymer, *The Palaeolithic Age* (Croom Helm, 1982).

113. M. Mussi, E. Mendez-Quintas, D. Barboni, H. Bocherens, R. Bonnefille, G. Briatico, D. Geraads, R. T. Melis, J. Panera, L. Pioli, A. Serodio Domínguez, S. Rubio Jara, A surge in obsidian exploitation more than 1.2 million years ago at Simbiro III (Melka Kunture, Upper Awash, Ethiopia). *Nat. Ecol. Evol.* **7**, 337–346 (2023).

114. J. Paige, C. Perreault, 3.3 million years of stone tool complexity suggests that cumulative culture began during the Middle Pleistocene. *Proc. Natl. Acad. Sci. U.S.A.* **121**, e2319175121 (2024).

115. D. A. Roe, Some Hampshire and Dorset handaxes and the question of ‘Early Acheulian’ in Britain. *Proc. Prehist. Soc.* **41**, 1–9 (1975).

116. J. Cook, R. Jacobi, “Observations on the Artefacts from the Breccia at Kent’s Cavern,” in *Stone Age Archaeology: Essays in Honour of John Wymer*, N. Ashton, F. Healy, P. Pettit, Eds., Oxbow Monograph 102, Lithics Study Society Occasional Paper 6 (Oxbow Books, 1998), pp. 77–89.

117. M. J. White, D. R. Bridgland, D. C. Schreve, T. S. White, K. E. H. Penkman, Well-dated fluvial sequences as templates for patterns of handaxe distribution: Understanding the record of Acheulean activity in the Thames and its correlatives. *Quat. Int.* **480**, 118–131 (2018).

118. R. Davis, N. Ashton, M. Hatch, P. G. Hoare, S. G. Lewis, Palaeolithic archaeology of the Bytham River: Human occupation of Britain during the early Middle Pleistocene and its European context. *J. Quat. Sci.* **36**, 526–546 (2021).

119. P. García-Medrano, C. Shipton, M. White, N. Ashton, Acheulean diversity in Britain (MIS 15-MIS11): From the standardization to the regionalization of technology. *Front. Earth Sci.* **10**, 917207 (2022).

120. A. Key, T. Lauer, M. Skinner, M. Pope, D. R. Bridgland, L. Noble, T. Proffitt, On the earliest Acheulean in Britain: First dates and in-situ artefacts from the MIS 15 site of Fordwich (Kent, UK). *R. Soc. Open Sci.* **9**, 211904 (2022).

121. D. A. Roe, British Lower and Middle Palaeolithic handaxe groups. *Proc. Prehist. Soc.* **34**, 1–82 (1968).

122. J. McNabb, Looking backwards, looking forwards: Evaluating the Roe handaxe methodology in the twenty-first century and the introduction of a new 'Roe-type' index. *Lithic Technol.* **47**, 183–202 (2022).

123. D. R. Bridgland, M. J. White, Fluvial archives as a framework for the Lower and Middle Palaeolithic: Patterns of British artefact distribution and potential chronological implications. *Boreas* **43**, 543–555 (2014).

124. D. R. Bridgland, M. J. White, Chronological variations in handaxes: Patterns detected from fluvial archives in north-west Europe. *J. Quat. Sci.* **30**, 623–638 (2015).

125. J. Orłowska, K. Cyrek, G. P. Kaczmarczyk, W. Migal, G. Osipowicz, Rediscovery of the Palaeolithic antler hammer from Biśnik Cave, Poland: New insights into its chronology, raw material, technology of production and function. *Quat. Int.* **665-666**, 48–64 (2023).

126. R. G. Klein, "Fully modern humans," in *Archaeology at the Millennium*, G. M. Feinman, T. Douglas Price, Eds. (Springer, 2001), pp. 107–135.

127. P. G. Chase, Tool-making tools and Middle Paleolithic behavior. *Curr. Anthropol.* **31**, 443–447 (1990).

128. J. Rosell, R. Blasco, J. F. Peris, E. Carbonell, R. Barkai, A. Gopher, Recycling bones in the Middle Pleistocene: Some reflections from Gran Dolina TD10-1 (Spain), Bolomor Cave (Spain) and Qesem Cave (Israel). *Quat. Int.* **361**, 297–312 (2015).

129. S. Lehnig, J. M. Hutson, E. Turner, A. Villaluenga, A. García-Moreno, G. Carver, S. Gaudzinski-Windheuser, Interpreting the Schöningen 13II-4 butchery sequence using the Harris Matrix. *J. Archaeol. Sci. Rep.* **36**, 102833 (2021).

130. P. Villa, G. Boschian, L. Pollarolo, D. Saccà, F. Marra, S. Nomade, A. Pereira, Elephant bones for the Middle Pleistocene toolmaker. *PLOS ONE* **16**, e0256090 (2021).

131. P. F. Cassoli, C. De Giuli, A. M. Radmilli, A. G. Segre, "Giacimento del Paleolitico inferiore a Malagrotta (Roma)," in *Atti XXIII Riunione Scientifica Istituto Italiano di Preistoria e Protostoria, Il Paleolitico inferiore in Italia, Firenze* , (Istituto Italiano di Preistoria e Protostoria, 1982), vol. 1982, pp. 531–549.

132. R. Rabinovich, O. Ackermann, E. Aladjem, R. Barkai, R. Biton, I. Milevski, N. Solodenko, O. Marder, Elephants at the middle Pleistocene Acheulian open-air site of Revadim Quarry, Israel. *Quat. Int.* **276-277**, 183–197 (2012).

133. G. Gvirtzman, M. Wieder, O. Marder, H. Khalaily, R. Rabinovich, H. Ron, Geological and pedological aspects of an Early-Paleolithic site: Revadim, Central Coastal Plain, Israel. *Geoarchaeology* **14**, 101–126 (1999).

134. M. H. Moncel, I. Biddittu, G. Manzi, B. Saracino, A. Pereira, S. Nomade, C. Hertler, P. Voinchet, J.-J. Bahain, Emergence of regional cultural traditions during the Lower Palaeolithic: The case of Frosinone-Ceprano basin (Central Italy) at the MIS 11-10 transition. *Archaeol. Anthropol. Sci.* **12**, 185 (2020).

135. I. Biddittu, A. G. Segre, "Utilizzazione dell'osso nel Paleolitico inferiore italiano." in *Atti XXIII Riunione Scientifica Istituto Italiano di Preistoria e Protostoria*, (Istituto Italiano di Preistoriae Protostoria, 1982), pp. 89–105.

136. A. Ascenzi, *Dives Anagnia: archeologia nella valle del Sacco* (L'Erma di Bretschneider, 1993), pp. 38–47.

137. A. Pereira, S. Nomade, M. H. Moncel, P. Voinchet, J. J. Bahain, I. Biddittu, C. Falguères, B. Giaccio, G. Manzi, F. Parenti, G. Scardia, V. Scao, G. Sottili, A. Vietti, Integrated

geochronology of Acheulian sites from the southern Latium (central Italy): Insights on human-environment interaction and the technological innovations during the MIS 11-MIS 10 period. *Quat. Sci. Rev.* **187**, 112–129 (2018).

138. A. M. Rodmilli, G. Boschian, *Gli scavi a Castel di Guido. Il più antico giacimento di cacciatori nell'Agro Romano* (ETS, 1996).

139. B. Mecozzi, I. Fiore, B. Giaccio, F. Giustini, S. Mercurio, L. Monaco, A. Argento, F. Bucci Casari degli Atti Di Sassoferrato, I. Caricola, C. Lemorini, F. Lucchini, I. Mazzini, M. R. Palombo, R. Sardella, A. Sposato, E. L. Spinapolice, F. Alhaique, From meat to raw material: The Middle Pleistocene elephant butchery site of Casal Lumbroso (Rome, central Italy). *PLOS ONE* **20**, e0328840 (2025).

140. D. Mania, U. Mania, "Bilzingsleben - *Homo erectus*, his culture and his environment. The most important results of research," in *Lower Palaeolithic Small Tools in Europe and The Levant*, J. M. Burdukiewicz, A. Ronen, Eds., B.A.R British Series (BAR Publishing, 2003), pp. 29–48.

141. E. Brühl, "The small flint tool industry from Bilzingsleben – Steinrinne," in *Lower Palaeolithic Small Tools in Europe the Levant*, J. M. Burdukiewicz, A. Ronen, Eds., B.A.R British Series, (BAR Publishing, 2003), pp. 49–63.

142. R. G. Bednarik, The Lower Paleolithic engravings of Bilzingsleben, Germany. *Encyclopedia* **4**, 695–708 (2024).

143. V. N. Stepanchuk, O. O. Naumenko, The earliest evidence of deliberate ivory processing dates back to around 0.4 million years ago. *Int. J. Osteoarchaeol.* **35**, e3403 (2025).

144. V. Dobosi, "Changing environment – Unchanged culture at Vértesszőlős, Hungary," in *Lower Palaeolithic Small Tools in Europe and the Levant*, J. M. Burdukiewicz, A. Ronen, Eds, British Archaeological Reports, International Series 1115 (Archaeopress, 2003), pp. 101–111.

145. P. Villa, A. P. Anzidei, E. Cerilli, "Bones and bone modifications at La Polledrara, a Middle Pleistocene site in Italy," in *The Role of Early Humans in the Accumulation of European*

Lower and Middle Palaeolithic Bone Assemblages, S. Gaudzinski, E. Turner, Eds. (Monographien des Romisch-Germanischen Zentralmuseum, 1999), vol. 42, pp. 197–206.

146. A. P. Anzidei, "Tools from elephant bones at La Polledrara di Cecanibbio and RebibbiaCasal de' Pazzi," *in The World Elephants: Proceedings of the First International Congress*, Rome, G. Cavarretta, P. Gioia, M. Mussi & M. R. Palombo, Eds. 16–20 October 2001 (Consiglio Nazionale delle Ricerche, Rome, 2001), pp. 415–418.

147. A. P. Anzidei, G. M. Bulgarelli, P. Catalano, E. Cerilli, R. Gallotti, C. Lemorini, M. Salvatore, M. R. Polombo, W. Pantano, E. Santucci, Ongoing research at the late Middle Pleistocene site of la Polledrara di Cecanibbio (Central Italy), with emphasis on human-elephant relationships. *Quat. Int.* **255**, 171–187 (2012).

148. C. Lemorini, E. Santucci, I. Caricola, A. Nucara, S. Nunziante-Cesaro, Life around the elephant in space and time: An integrated approach to study the human-elephant Interactions at the Late Lower Paleolithic Site of La Polledrara di Cecanibbio (Rome, Italy). *J. Archaeol. Method Theory* **30**, 1–49 (2023).

149. E. Cerilli, I. Fiore, Natural and anthropic events at La Polledrara di Cecanibbio (Italy, Rome): Some significant examples. *Alp. Mediterr. Quat.* **31**, 55–58 (2018).

150. M.-A. Julien, B. Hardy, M. C. Stahlschmidt, B. Urban, J. Serangeli, N. J. Conard, Characterizing the Lower Paleolithic bone industry from Schöningen 12 II: A multi-proxy study. *J. Hum. Evol.* **85**, 264–286 (2015).

151. M. Domínguez-Rodrigo, "Artefactos" óseos en Torralba y Ambrona: Estudio de piezas sobre hueso post-craneal depositadas en el Museo Arqueológico Nacional. *Zona Arqueológica* **5**, 282–287 (2005).

152. P. Villa, F. d'Errico, Las puntas de marfil de Torralba y Ambrona. *Zona Arqueológica* **5**, 288–304 (2005).

153. A. Pineda, P. Saladié, The Middle Pleistocene site of Torralba (Soria, Spain): A taphonomic view of the Marquis of Cerralbo and Howell faunal collections. *Archaeol. Anthropol. Sci.* **11**, 2539–2556 (2019).

154. G. Wei, C. He, Y. Hu, K. Yu, S. Chen, L. Pang, Y. Wu, W. Huang, W. Yuan, First discovery of a bone handaxe in China. *Quat. Int.* **434**, 121–128 (2017).
